# Subcortical correlates of developmental language disorder: more than the neostriatum

**DOI:** 10.1093/braincomms/fcaf493

**Published:** 2025-12-17

**Authors:** Gabriel J Cler, Salomi S Asaridou, Nilgoun Bahar, Saloni Krishnan, Harriet J Smith, Hanna E Willis, Máiréad P Healy, Kate E Watkins

**Affiliations:** Department of Experimental Psychology, Centre for Integrative Neuroimaging, University of Oxford, Oxford OX1 3EL, UK; Department of Speech and Hearing, University of Washington, Seattle, WA 98105, USA; Department of Experimental Psychology, Centre for Integrative Neuroimaging, University of Oxford, Oxford OX1 3EL, UK; Department of Experimental Psychology, Centre for Integrative Neuroimaging, University of Oxford, Oxford OX1 3EL, UK; Department of Neurology, Dyslexia Center, UCSF, San Francisco 94158, USA; Department of Experimental Psychology, Centre for Integrative Neuroimaging, University of Oxford, Oxford OX1 3EL, UK; Division of Psychology and Language Sciences, Department of Language and Cognition, University College London, London WC1N 1PF, UK; Department of Experimental Psychology, Centre for Integrative Neuroimaging, University of Oxford, Oxford OX1 3EL, UK; Division of Psychology and Language Sciences, Department of Speech Hearing and Phonetic Sciences, University College London, London WC1H 0AP, UK; Department of Experimental Psychology, Centre for Integrative Neuroimaging, University of Oxford, Oxford OX1 3EL, UK; Department of Experimental Psychology, Centre for Integrative Neuroimaging, University of Oxford, Oxford OX1 3EL, UK; Department of Psychology, University of Cambridge, Cambridge CB2 3EB, UK; Department of Experimental Psychology, Centre for Integrative Neuroimaging, University of Oxford, Oxford OX1 3EL, UK

**Keywords:** developmental language disorder, subcortical grey matter, volumes, paediatric, MRI

## Abstract

Developmental language disorder (DLD) is a common neurodevelopmental disorder that affects receptive and expressive language skills. In contrast to the wealth of evidence on acquired language disorders, we understand relatively little about the neural underpinnings of DLD. A recent meta-analysis across different types of structural brain analyses in DLD highlighted consistent anatomical differences in the anterior striatum, with other subcortical structures relatively spared. These findings are consistent with predictions from the procedural circuit deficit hypothesis (PCDH), namely that the anterior neostriatum differs in structure and function in DLD, whereas medial temporal lobe structures are unaffected and may act in a compensatory manner. Here, in a case–control study with a larger sample size than previous studies, we evaluated volume and microstructure of subcortical grey matter structures using T1-weighted images and diffusion imaging. Our predictions were partly in accord with those of the PCDH and the findings of the meta-analysis. Neuroimaging and behavioural measures were acquired in 156 children and adolescents (54 DLD; 74 typically developing (TD); 28 with a history of language difficulties) aged 10:0–15:11 years. As predicted by the PCDH, there were significant differences in the DLD group in volume and microstructure of the neostriatum (caudate nucleus, putamen). However, in contrast to our prediction, there were also significantly smaller structures in the DLD group across other subcortical structures evaluated: globus pallidus, thalamus and hippocampus. The hippocampal difference is of particular interest as it is hypothesized in the PCDH to be spared in DLD. Microstructural measures (diffusion tensor imaging and neurite orientation dispersion and density imaging) revealed differences in the caudate nucleus, thalamus and hippocampus. Multivariate machine learning analyses highlighted the relationship between the hippocampus and language skills but only in the TD cohort. We conclude that the subcortical correlates of DLD are in fact not limited to the neostriatum and represent important areas of further inquiry.

## Teaser

Cler *et al*. report an analysis of subcortical nuclei in a large sample with a range of language abilities (*N* = 156). Smaller neostriatal volumes were associated with DLD as predicted. Unexpectedly smaller volumes were additionally reported for the hippocampus, pallidum and thalamus.

## Graphical Abstract

**Graphical Abstract:**
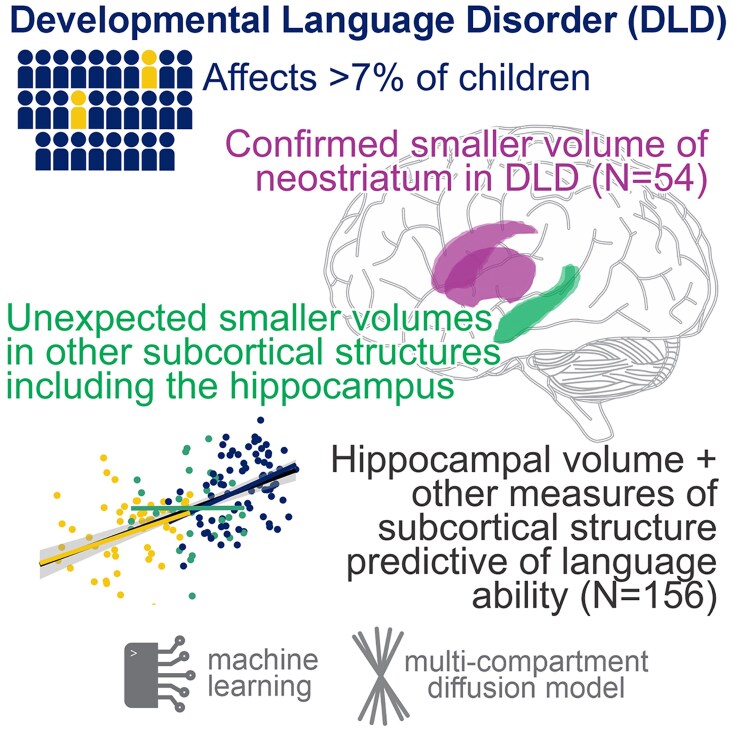

## Introduction

An estimated 7.6% of the population struggles to learn their first language^1^ due to a neurodevelopmental disorder called developmental language disorder (DLD). DLD is defined as an unexplained language problem that endures into middle childhood and beyond and has a significant impact on social and educational function.^2^ The behavioural profile is heterogeneous. Children with DLD often have deficits in grammar, vocabulary and literacy along with non-linguistic differences in motor control and sequence or statistical learning (see review^3^). Unlike in acquired language disorders such as aphasia, it is unclear what brain differences give rise to these developmental challenges in language learning.

A well-known unifying theory of DLD, the procedural circuit deficit hypothesis (PCDH), suggests that DLD is explained by abnormalities in the structures underlying procedural memory, whereas declarative memory and its underlying structures are spared.^4,5^ Procedural memory includes skills and habits often acquired and accessed implicitly, which are assessed by task performance rather than explicit or verbalizable recall.^6^ The declarative memory system, alternately, involves the conscious recall of facts and events, allowing us to explicitly remember knowledge and experiences.^7^ The procedural system is thought to be important for learning and remembering grammar, while the declarative system is thought to be important for learning and remembering vocabulary.

In the PCDH, the procedural system is defined not strictly behaviourally but as those functions that rely on the basal ganglia and its associated circuitry.^4^ The basal ganglia include the neostriatum (caudate nucleus and putamen), globus pallidus, substantia nigra and subthalamic nucleus. Both the indirect and direct pathways of these circuits are relayed through the thalamus on the way to the cortex. The nucleus accumbens is part of the ventral striatum. Accordingly, declarative memory is defined as that which relies on medial temporal lobe structures such as the hippocampus, amygdala and parahippocampal cortex. In this framework, the neurobiological basis of DLD is these procedural memory deficits tied to basal ganglia differences, and the medial temporal lobe should be unaffected in DLD and could thus even compensate for procedural learning impairments.^4^

A recent meta-analysis^8^ combined findings from studies in DLD that separately investigated subcortical, cortical or cerebellar structure, and those that looked at functional MRI. The results supported the main predictions of the PCDH, finding that the most consistent differences across studies of DLD were in the striatum, specifically the anterior neostriatum (head of the caudate nucleus and anterior putamen). This finding represents a significant advance in understanding the neuroanatomy of DLD, suggesting that despite the heterogeneity in the behavioural profile, consistent abnormalities are detectable in the neostriatum but not the medial temporal lobe. The authors argue that this should lead to a focus on the basal ganglia, opening the door to exploring pharmacological interventions that improve procedural memory, and suggest that spared declarative memory could be targeted to provide a compensatory role in interventions. The null results in medial temporal lobe structures are pivotal to the theory, but the methods used in the meta-analysis may have been insensitive to small effects since it considered only binary ‘significant’ and ‘non-significant’ effects across several underpowered studies, which will inherently fail to yield robust null effects.

It is critical to note that only seven previous studies evaluated subcortical grey matter volume in DLD (see Supplementary Table 1).^9-15^ Other papers looking at structural data were either case studies^16,17^ or focused on selected cortical regions of interest.^18,19^ When looked at separately, the results of the studies evaluating subcortical structure in DLD varied substantially, as did the age ranges studied (from 5 years old to adulthood), sample sizes (minimum of 10 and maximum of 36 with DLD) and methods used. The most common difference reported was a smaller caudate nucleus in DLD^13,15-17^ although one study found that this structure was larger when considering only younger children with DLD.^15^ Two studies found that the putamen was larger in DLD, but these differences did not survive correction for multiple comparisons in one report^14^ and were only evident when a correction for intracranial volume (ICV) was used in the other.^13^

Whether to correct for intracranial or whole-brain volume when measuring subcortical volumes in developmental disorders is an important consideration. Since these volumes combine tissues with distinct developmental trajectories, group differences in relative and absolute volumes of subcortical structures may be differentially sensitive to more global or more local developmental mechanisms. Whole-brain volume was found to be smaller in DLD in one study (young adults),^13^ larger in another (children aged 5–11)^11^ and unaffected in a third (children/adolescents aged 8–17; no difference in total grey matter volume was reported in the paper but ICV also did not differ between groups [personal communication]).^9^ In the study of 12 adults with DLD who had significantly smaller whole-brain volume relative to controls, correcting for whole-brain volume substantially affected the results.^13^ Absolute volumes of the caudate nucleus and thalamus were smaller in DLD relative to controls but when corrected for whole-brain volume these differences disappeared and the corrected volumes of all the remaining structures (putamen, nucleus accumbens, and hippocampus; right globus pallidus) were larger in the DLD group than in controls.^13^

To address the issue of small samples and inconsistent methods for measuring subcortical volumes, we analysed data from the Oxford Brain Organisation in Language Development (OxBOLD) Study. This is a large, well-characterized sample of children and adolescents aged 10–15 years with a range of language learning abilities.^20-22^ The age range was selected to be overlapping with the previous studies of subcortical structure, and to ensure compliance with the brain imaging and behavioural data collection, which took 1 and 2 h, respectively. Stakeholders, including speech-language therapists/pathologists, educators, children and young people with DLD and their caregivers, have expressed interest in outcomes in this age range.^23^ Individuals in the study were assigned to one of three groups based on previous concerns about speech or language development and performance on tests of receptive and expressive vocabulary, sentence processing and narrative. Those with a history of speech and languageconcerns were assigned to the DLD group if they showed low language ability on at least two tests.^24^ Another smaller group had a history of speech and language difficulties, including a diagnosis of DLD but did not perform poorly on two or more language tests (HSL group). The typically developing (TD) group had no history of speech or language difficulties and did not perform poorly on two or more language tests (see^25^). Brain volumes were measured from structural MRI T1-weighted scans using FreeSurfer, which has been used extensively in other imaging studies of subcortical volumes.^26-28^

In accordance with the PCDH and other theoretical accounts of DLD, we predicted that a macrostructural analysis of subcortical structures would reveal smaller volumes of structures of the neostriatum (caudate nucleus, putamen) in DLD.^8,29^ Importantly, based on the PCDH, we further predicted that medial temporal lobe (hippocampus and amygdala) volumes would not be lower in DLD since these structures support declarative memory functions that could be used to compensate for the procedural memory impairment. Please note that to be consistent with^8^ and reduce confusion when referring to the anterior portions of the dorsal striatum (caudate nucleus and putamen), here we use ‘neostriatum’ synonymously with the dorsal striatum.

It is worth noting that in development, both smaller and larger volumes of brain structure would indicate atypical maturation. Smaller volumes might reflect smaller cell number and size (for both neurons and glia), or lower connectivity and myelination. Larger volumes might reflect delayed maturation due to an initial overproliferation of dendrites, less ‘pruning’ of connections and less ‘whitening’ of grey matter from myelin, lower packing density or more cerebro-spinal fluid in tissue. To understand the potential cellular mechanisms underlying any gross volume differences (macrostructure), we included an analysis of tissue characteristics within those structures (microstructure) using diffusion imaging metrics obtained in the same cohort. Our previous whole-brain analysis of the same cohort revealed abnormal levels of myelin in the dorsal striatum bilaterally and left hemisphere cortical language regions in DLD.^30^ We therefore expected the neostriatum to also show abnormal microstructure in DLD based on fractional anisotropy (FA) and mean diffusivity (MD), which measure the directionality and magnitude of water diffusion, respectively. FA is typically low in grey matter and indexes cell density, organization and iron; MD in grey matter is additionally sensitive to oedema and cell death. Our diffusion sequence allowed further investigation of microstructure, modelling intra- and extra-cellular and isotropic diffusion, which index diffusion within axons and dendrites, dispersion of fibres and the contribution from cerebro-spinal fluid, respectively. Finally, we used a multivariate approach across data from all 156 participants to determine the best combination of macro- and microstructural predictors of language performance. Even in the absence of significant group differences in individual measures, evidence that macro- or micro-structural features of the basal ganglia or medial temporal lobe structures explain variance in language learning would provide support for predictions arising from the PCDH.

## Materials and methods

### Participants

Participants were included if they had grown up in the UK speaking English and had normal hearing. During recruitment, telephone screening with a caregiver determined eligibility for the study applying the following exclusion criteria: (i) a diagnosis of another developmental disorder such as Down syndrome or Williams syndrome; (ii) a history of neurological impairments or neurological disorders such as epilepsy; (iii) a diagnosis of autism spectrum disorder or attention deficit hyperactivity disorder; (iv) a score above 7 (i.e. in the clinical range) on the hyperactivity subscale of the Strengths and Difficulties Questionnaire^31^; (v) a score above 15 (suggestive of autism spectrum disorder) on the Social Communication Questionnaire—Lifetime^32^ or (vi) a contraindication to MRI.^25,33-40^

175 participants were recruited and underwent a 2-h comprehensive neuropsychological test battery and a 1-h MRI scan session. All participants passed audiometric screening at 25 dB at 500, 1000, and 2000 Hz in the better ear. Data in 15 of the 175 participants were excluded: three because they had a non-verbal IQ < 70 (assessed with the WISC-IV Matrix Reasoning and Block Design Tests)^41^; a further three who were found not to have grown up speaking English before the age of 5; one because of poor performance on language tests but no history of speech and language problems; two because they did not complete the behavioural testing; three because they did not complete the MRI scan; three due to an incidental finding on the MRI scan. Quality control on MRI led to an additional four scans being excluded (see below), leading to a final sample of 156 participants.

### Behavioural testing

As part of an extensive behavioural battery, participants completed several language tests that were used to determine language ability and group classification: the Test for Reception of Grammar (TROG)^36^; the Recalling Sentences subtest of the Clinical Evaluation of Language Fundamentals—4th Edition (CELF)^37^; the Expression, Reception and Recall of Narrative Instrument (ERRNI)^38^; the Receptive One-Word Picture Vocabulary Test—4th Edition (ROWPVT)^39^ and the Expressive One-Word Picture Vocabulary Test—4th Edition (EOWPVT).^40^ Participants were categorized as TD if they had no history of speech and language problems and no more than one language test score lower than 1 SD below its normative mean.^25^ Participants were categorized as having DLD if they presented with a history of speech and language problems and scored at least 1 SD below the normative mean on two or more language tests.^33-35^ A third group of participants was identified with a history of speech and language problems but who did not score below 1SD lower than the mean on more than one test. This resulted in 54 participants in the DLD (mean age = 12.43 years; 15 female), 74 TD (mean age = 12.6 years; 33 female) and 28 HSL (mean age = 12.4 years; 5 female) groups.

Caregivers completed the Children’s Communication Checklist-2 (CCC-2^42^), a screening questionnaire used to assess social communication difficulties and their potential impact on daily life in children with language disorder.

The remaining tests in the behavioural battery provided measures of non-word repetition,^1,43^ auditory short-term, working memory and episodic memory (Digit Span Forward and Backward and the Word Lists subtests of the Children’s Memory Scale^44^), non-verbal reasoning (Block Design, Matrix Reasoning and Coding subtests of the Wechsler Intelligence Scale for Children, 4th Edition^45^), reading (Test of Word Reading Efficiency; TOWRE^46^), oromotor skills (Oromotor Sequences subtest of the NEuroPSYchology test^47^) and manual dexterity (Purdue Pegboard Test^48,49^).

To summarize performance across this extensive battery of behavioural tests, improve the reliability of the measures and avoid running separate analyses for each of the many scores, we derived language and memory factor scores for each participant. A pre-registered^20^ two-factor hybrid exploratory-confirmatory approach to factor analysis identified the best weighted combination of language and memory measures to give a language factor score and a memory factor score. Anchor measures for these factors were the expressive vocabulary score from the EOWPVT and list learning standard score from the Children’s Memory Scale, respectively; correlations between scores were modelled. Performance on the CELF sentence recall task, oromotor tasks, expressive vocabulary and non-word repetition contributed the largest weightings to the language factor. Accordingly, the language factor was highly correlated with the CELF sentence recall score (Pearson’s correlation = 0.94). Sentence repetition requires morphosyntactic analysis and reconstruction of the meaning of sentences. It depends on both language and memory systems, and it is thought to be a sensitive marker for DLD.^50^ Scores for immediate and delayed recall of the word lists and word list recognition contributed the largest weightings to the memory factor, reflecting long-term memory performance rather than capturing variance in short-term or working memory, which contributed minor weightings to the language factor score.

We compared the TD and DLD groups on the brain measures and used the language and memory factors in the multivariate analyses of the whole cohort, which included the HSL group.

### MRI acquisition

Images were acquired on a Siemens Prisma 3T scanner with a 32-channel headcoil. The T1-weighted scans were MPRAGE (magnetization prepared low-angle spoiled gradient echo, TR 1900 ms, TE 3.97 ms, flip angle 8°, field of view 174 × 192 × 192 mm), with 1 × 1 × 1 mm isometric voxels. The acquisition took 5 min and 30 s, during which children watched a movie. Diffusion-weighted MRI was acquired with the UK Biobank Project protocol^51^: 2 × 2 × 2 mm voxels with shells at *b* = 1000, 2000 s/mm^2^; multiband factor 3; 50 distinct diffusion directions in each shell. B0 images with typical and reverse phase-encoding directions were also acquired.

### Morphological analysis

Automatic segmentation of the subcortical structures was performed with FreeSurfer 7.2.0 (http://surfer.nmr.mgh.harvard.edu) as part of the ‘recon-all’ pipeline. This procedure includes segmentation of the subcortical white matter and deep grey matter.^52,53^ Automated quality control was implemented with Qoala-T^54^: Fifty-one scans were recommended for visual inspection, with nine recommended as possible exclusions. All but four scans were retained after visual inspection.^22^ Volumes of each subcortical region mask were exported for statistical analysis using the Stats2Table.R function modified for FreeSurfer 7, available as part of Qoala-T (https://github.com/Qoala-T/QC/blob/master/Scripts/Stats2Table/Stats2Table_fs7.R).

#### Intracranial volume

ICV was extracted from FreeSurfer as ‘EstimatedTotalIntraCranialVol’. Possible group differences in ICV were evaluated with a linear model including covariates of group, age and sex and their interactions (ICV = Group*Age*Sex).

#### Subcortical structure volume

Statistical analysis was performed in R (v4.3.2). Volumes were normalized for ICV via the residual approach, e.g.^55^ In brief, a linear regression is run between each set of volumes (e.g. all left putamen volumes) and all ICVs to get the slope of the relationship. Then, the volumes are normalized as follows: the slope of the relationship between the volume of a structure and ICV was calculated for all participants in the study and used to adjust each individual’s structure volume for their ICV relative to the study mean ICV using the following formula.


$$V_{i\_\mathrm{adj}}=V_{i}-\mathrm{slope}*(\mathrm{IC}V_{p}-\mathrm{mean}\;\mathrm{ICV})$$

in which *V_i_* is the volume of one participant’s subcortical structure, ICV_p_ is that participant’s ICV and mean ICV is the average across all participants.

Corrected volumes that were ±2.5 SDs from the mean were removed (38 volumes total out of 156 participants × 7 structures × 2 hemispheres = 2184 data points; 1.7% removed). Of these 38 volumes, 20 were from the left hemisphere, 14 were from the DLD group, 15 from the HSL group and 9 from the TD group. Twenty-five were above the mean + 2.5 SDs limit, and the rest were below the mean −2.5 SDs. All structures had some outliers: nucleus accumbens (9), hippocampus (6), amygdala (5), pallidum (5), thalamus (5), caudate nucleus (4) and putamen (4). Six participants had data removed for two structures (in three these were the left and right measurements of the same structure); two HSL participants had more than two structure volumes removed; in the remaining 16, only one volume was removed.

##### Statistical analysis of volumetric data

Linear mixed models were run for each pair of structures: caudate nucleus (CN), putamen (Put), nucleus accumbens (NA), pallidum (Pall), thalamus (Thal), hippocampus (Hipp) and amygdala (Amyg). Simple models included hemisphere, group and their interaction to predict volume, with participant as a random factor (volume ∼ hemisphere + group + group*hemisphere + (1|participant)). Alternative models were run with additional covariates: hemisphere, group, their interaction, plus age, handedness, sex and ICV, with participant as a random factor (volume ∼ hemisphere + group + group*hemisphere + age + handedness + sex + ICV + (1|participant)). The pattern of results was similar between simple and alternative models, but alternative models gave statistically better fits via likelihood ratio tests on most structures, so those alternative models are presented here. Models were constructed in R with *lmer*. Results were corrected for multiple comparisons using a false discovery rate (FDR; Benjamini–Hochberg correction) of *q* < 0.05 across all subcortical structures and model parameters.^56^

Given that the results of a previous study differed considerably with and without correcting for ICV,^13^ we also reran analyses with different types of ICV correction. First, we used no ICV residual correction and just included ICV in our statistical models. Then, we removed ICV entirely from the model but still included age and sex. Finally, we reran the models without age, sex or ICV, and without ICV correction. In all cases, the pattern of results held, so only models of volumes corrected for ICV and with ICV included in the model are presented here.

#### Microstructural analysis

Diffusion metrics were analysed in grey matter to further characterize possible tissue microstructural differences in the same subcortical structures. Diffusion imaging was pre-processed with the FMRIB Software Library v6.0 (FSL).^57^ Images were corrected for susceptibility distortion with *topup*^58^ and for eddy currents and head motion with *eddy.*^59^ Diffusion tensor imaging (DTI) parameters were estimated using the *b* = 1000 shell with *dtifit*. FA is a measure of directional diffusion and is the highest in white matter voxels where diffusion is impeded by structures such as myelinated axons. In grey matter, FA is affected by packing density, structure and organization of neuronal processes and iron concentration. MD is another measure of the diffusion of water molecules in brain tissue, and high diffusivity would indicate a less compact structure.

Microstructure was further examined with neurite orientation dispersion (OD) and density imaging models (NODDI),^60^ which reduces the effects of free water (i.e. CSF) measurements. In subcortical grey matter, contrast-to-noise ratios have been shown to be higher in NODDI than typical DTI parameters,^61^ and NODDI parameters have recently been shown to be sensitive to differences in subcortical grey matter,^62,63^ in contrast to typical diffusion metrics that are most often used in white matter. NODDI protocols model diffusion data in three compartments: intra-cellular, extra-cellular and CSF, and derive measures of (i) the amount of diffusion that is isotropic (e.g. CSF; *f*_iso_), (ii) how much of the remaining diffusion is within a neurite (axons, dendrites) rather than outside of neurites (*f*_intra_) and (iii) how dispersed the fibres are (orientation dispersion; OD). NODDI parameters were estimated using both *b* = 1000 and 2000 shells with the CUDA diffusion modelling toolbox (cuDIMOT^64^) using the Watson model and Markov Chain Monte Carlo search.

Masks from FreeSurfer were linearly registered to individual diffusion space with *flirt*. Masks were eroded using a 4-mm box kernel to minimize partial volume effects and then applied to individual images of FA, MD and NODDI parameters. Average values were calculated within each mask and exported for statistical analysis. As with volume measures, FA and MD values ±2.5 SDs from the mean were removed (78 measurements total out of 156 participants × 7 structures × 2 hemispheres × 2 metrics = 4368 data points; 1.8% removed).

##### Statistical analysis of diffusion data

FA and MD were evaluated in each structure with linear mixed effect models with factors of hemisphere, group, their interaction, plus age, handedness and sex, with participant as a random factor (e.g. FA = hemisphere + group + group*hemisphere + age + handedness + sex + (1|participant)). To further explain microstructural differences that might underlie FA differences, Pearson’s correlations were run on the FA against each NODDI parameter (*f*_iso_, *f*_intra_, OD) within those structures with significantly different FA and the hippocampus.^65^ We also ran the same linear mixed effect models as above with all structures and all parameters (see Supplementary Tables 7–9).

### Multivariate analysis of brain structure and behaviour

Volumetric and diffusion analyses were combined in a multivariate analysis to explore the relationship of brain measures to language proficiency in all participants, across TD and DLD participants, and including the HSL group. Language proficiency was based on the language factor score, a continuous summary measure derived from a factor analysis of the behavioural measures, including results on tests of receptive and expressive vocabulary, grammar, narratives, non-word repetition and memory as detailed above.

We used machine learning (LASSO)^66^ to select relevant features among brain measures (volumes including ICV, OD, *f*_intra_, *f*_iso_) and demographic variables (age, sex, handedness) to predict language performance with a general linear model. NODDI parameters were selected rather than FA because highly correlated predictors make LASSO regression more unstable; FA is highly correlated with OD and *f*_intra_, as FA is affected by both the dispersion of fibres and the amount of fibres in an area. Models were constructed in R with *glmnet* with alpha = 1 (full LASSO regression) and 10 cross-validation folds. The stability of feature selection was also assessed across the 10-fold cross-validation. Models were built across groups and then within each group to examine the features selected to predict language performance.

## Results

### Behaviour

Data were available in 156 participants, 74 TD, 54 DLD and 28 HSL. The HSL group included participants with a history of speech and language concerns, a necessary inclusion criterion for the DLD group also, but did not meet criteria for inclusion in the DLD group due to better performance on language tests during the behavioural testing (i.e. they did not score 1SD or more below the mean on more than one test). Details of these groups including summaries of their performance on neuropsychological tests are provided in Table 1 and were previously reported.^22^ The groups did not differ in mean age or distribution of handedness. There were more males than females in the DLD and HSL groups compared with the TD group. The DLD group had lower average scores than TD on all measures of language, memory, non-verbal reasoning, reading, motor skills and on the communication checklist. Interestingly, the performance of the HSL group was between that of the TD and DLD groups on all tests (see Table 1 for details).

**Table 1:** Group demographics and test scores.

	TD (*n* = 74)	DLD (*n* = 54)	HSL (*n* = 28)	*P* value	Group differences
Demographics					
Mean age (years)	12.6 ± 0.2	12.4 ± 0.3	12.4 ± 0.3	0.785-	–
Age range (years; months)	10;0–15;7	10:0–15;11	10;5–15;7	–	–
Sex (M:F)	41:33	39:15	23:5	**0.019**	TD < DLD, HSL
Handedness (R:L)	63:11	46:8	24:4	0.997	–
Language					
ROWPVT-4	128.4 ± 1.9	100.1 ± 2.1	122 ± 2.9	**<0.001**	DLD < TD, HSL
EOWPVT-4	117.9 ± 1.7	92.4 ± 1.7	108.3 ± 2.5	**<0.001**	DLD < HSL < TD
TROG-2	105.2 ± 0.9 (*n* = 73)	80.9 ± 1.8 (*n* = 53)	98.9 ± 1.6	**<0.001**	DLD < HSL < TD
CELF-4 Recalling Sentences	11.6 ± 0.3 (*n* = 70)	4.9 ± 0.4	9 ± 0.6	**<0.001**	DLD < HSL < TD
ERRNI					
Initial Recall	100.1 ± 1.5	83.7 ± 1.7	96.3 ± 2.1	**<0.001**	DLD < TD, HSL
Delayed Recall	104.4 ± 1.3	85.1 ± 1.7	99.1 ± 1.9	**<0.001**	DLD < TD, HSL
Comprehension	106.7 ± 1.6	93.2 ± 2.1	101.9 ± 1.9	**<0.001**	DLD < TD, HSL
Non-word repetition (max. 30)	26.3 ± 0.3 (*n* = 71)	17.8 ± 0.8 (*n* = 53)	22.6 ± 0.7	**<0.001**	DLD < HSL < TD
Language summary measure^20^	0.8 ± 0.1	−1.0 ± 0.1	0.0 ± 0.1	**<0.001**	DLD < HSL < TD
Memory					
CMS					
Digit span forward	11.5 ± 0.3	5.8 ± 0.4	7.5 ± 0.6	**<0.001**	DLD, HSL < TD
Digit span backward	11.8 ± 0.3	7.2 ± 0.5	8.5 ± 0.6	**<0.001**	DLD, HSL < TD
Word list immediate recall	10.1 ± 0.4	6.2 ± 0.4 (*n* = 52)	8.4 ± 0.5	**<0.001**	DLD < HSL < TD
Word list delayed recall	10.2 ± 0.4 (*n* = 73)	7.3 ± 0.5 (*n* = 51)	9.8 ± 0.6	**<0.001**	DLD < TD, HSL
Word list delayed recognition	8.6 ± 0.4 (*n* = 73)	6.6 ± 0.5	7.4 ± 0.6	**0.003**	DLD, HSL < TD
Memory summary measure^20^	0.5 ± 0.1	−0.8 ± 0.1	0.0 ± 0.1	**<0.001**	DLD < HSL < TD
Non-verbal reasoning					
WISC-IV					
Block design	13.3 ± 0.2	9.8 ± 0.4	12.8 ± 0.4	**<0.001**	DLD < TD, HSL
Matrix reasoning	11.2 ± 0.3	7.6 ± 0.4	9.9 ± 0.5	**<0.001**	DLD < TD, HSL
Coding	9.6 ± 0.3 (*n* = 71)	5.9 ± 0.4	6.5 ± 0.4	**<0.001**	DLD, HSL < TD
Reading					
TOWRE-2					
Sight word efficiency	106 ± 1.3	82.6 ± 1.9	89.1 ± 2.6	**<0.001**	DLD, HSL < TD
Phonemic decoding efficiency	112.2 ± 1.6	81.3 ± 2.2	87.1 ± 2.5	**<0.001**	DLD, HSL < TD
motor					
NEPSY oromotor sequencing (max. 70)	60.5 ± 0.9	41.4 ± 1.5	53.4 ± 1.9	**<0.001**	DLD < HSL < TD
Purdue Pegboard					
Pegs moved with dominant hand (z-score)	−0.4 ± 0.1	−1.5 ± 0.2	−0.9 ± 0.2	**<0.001**	DLD < HSL < TD
Pegs moved with non-dominant hand (z-score)	0.0 ± 0.1	−1.1 ± 0.2	−0.8 ± 0.2	**<0.001**	DLD < HSL < TD
Mean difference (dominant − non-dominant)	0.6 ± 0.1	0.6 ± 0.2	0.6 ± 0.2	**<0.001**	DLD < HSL < TD
CCC-2					
GCC (scaled score with mean = 80)	85.3 ± 1.7	35.9 ± 2.7 (*n* = 53)	48.4 ± 4.4	**<0.001**	DLD < HSL < TD

Abbreviations: ROWPVT = Receptive One-Word Picture Vocabulary Test; EOWPVT = Expressive One-Word Picture Vocabulary Test; TROG = Test for Reception of Grammar; CELF = Clinical Evaluation of Language Fundamentals; ERRNI = Expression, Reception and Recall of Narrative Instrument; CMS = Children's Memory Scale; WISC = Wechsler Intelligence Scale for Children; TOWRE = Test of Word Reading Efficiency; NEPSY = A Developmental Neuropsychological Assessment; CCC = Children's Communication Checklist; GCC—General Communication Composite score.

Standard scores with means of 100 and SD of 15 or of 10 with SD of 3 are provided unless otherwise indicated. Bold values indicate *P* < 0.05.

**Figure 1:**
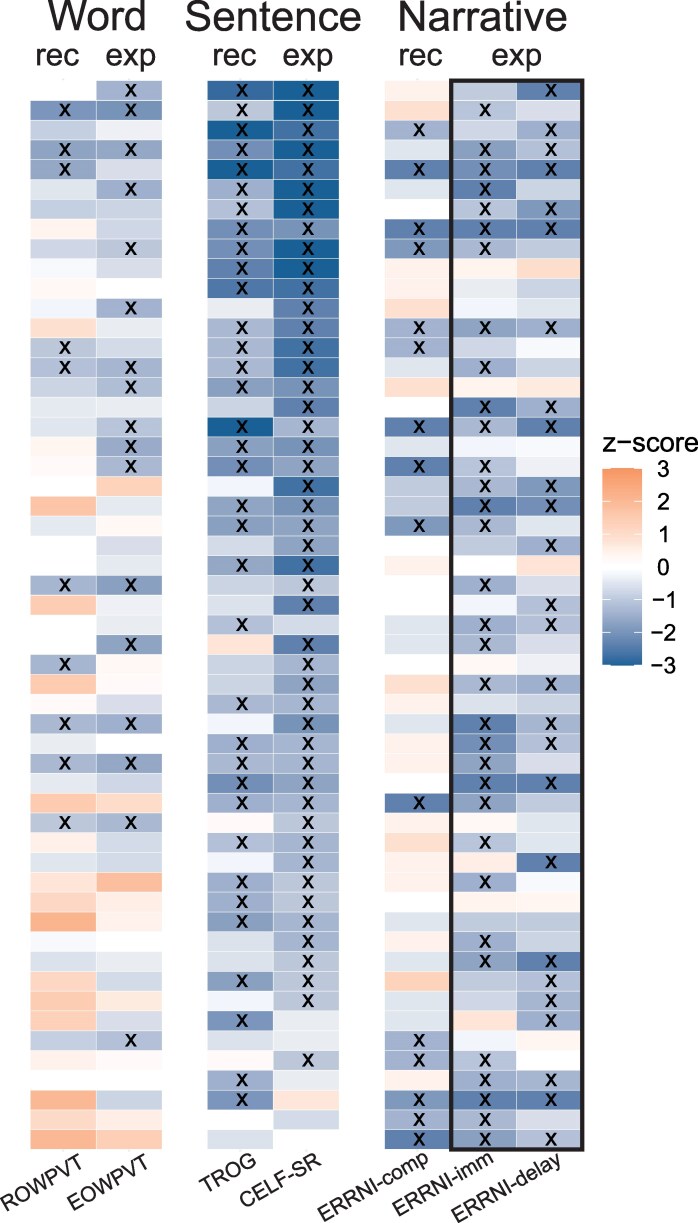
**Visual summary of language test performance within the DLD group.** Columns show individual participant performance on tests across word, sentence and narrative processing for both expressive and receptive tasks. Orange shows areas of relative strength and blue relative weakness. For the word level, the receptive task was the ROWPVT and the expressive task the EOWPVT. For the sentence level, the receptive task was the TROG, and the expressive task was the CELF sentence recall task. For the narrative level, the receptive task was the ERRNI comprehension score, and there were two expressive tasks, the ERRNI-immediate and ERRNI-delay tasks. All were z-scored according to the standard mean and SD from normative tests. Test performance at or below −1SD is marked with an ‘x’. Note that all participants must have had at least two test scores at or below −1SD, but that the ERRNI-immediate and ERRNI-delay could not be the only two tests they failed. Participants are ordered by the continuous summary language proficiency measure described in Krishan *et al*. 2021, with the lowest proficiency at the top. Each row is one participant in the DLD group; each box is the performance on one task (*N* = 1). Abbreviations: rec = receptive language test; exp = expressive language test; DLD = developmental language disorder; ROWPVT = Receptive One-Word Picture Vocabulary; EOWPVT = Expressive One-Word Picture Vocabulary Test; TROG = Test for Reception of Grammar; CELF-SR = Clinical Evaluation of Language Fundamentals—Sentence Recall; ERRNI = Expression, Reception and Recall of Narrative Instrument.

As noted in the introduction, the behavioural profile in DLD is a heterogeneous disorder. Along with a history of speech and language concerns, participants in the DLD group had at least two low scores on six measures of language. We summarized the performance of participants in the DLD group across these six measures in Fig. 1. It is notable that of the 54 participants in the DLD group, 51 of them score below −1SD on at least one of two sentence-level grammatical tests (CELF-sentence recall; TROG). The three participants who did not show this difficulty had low scores on at least one measure of narrative processing. Receptive and expressive vocabulary were not common areas of difficulty, and scores were above the mean in some participants.

### Volumetric differences

#### Intracranial volume

The TD and DLD groups did not differ in ICV (TD M = 1339.6 cc, SD = 136.5 cc; DLD M = 1323.6 cc, SD = 164.6 cc). As expected, ICV was larger in boys (significant main effect of sex: β = 2.00, *P* = 0.048) and increased more steeply with age in the boys compared with girls (significant interaction effect of sex × age: β = 2.71, *P* = 0.008; Supplementary Table 2). The volumes of subcortical structures analysed and reported here were corrected for ICV using linear regression (see methods). ICV was also included as a covariate in the analyses described below along with age, handedness and sex.

#### Subcortical structure volume

**Figure 2:**
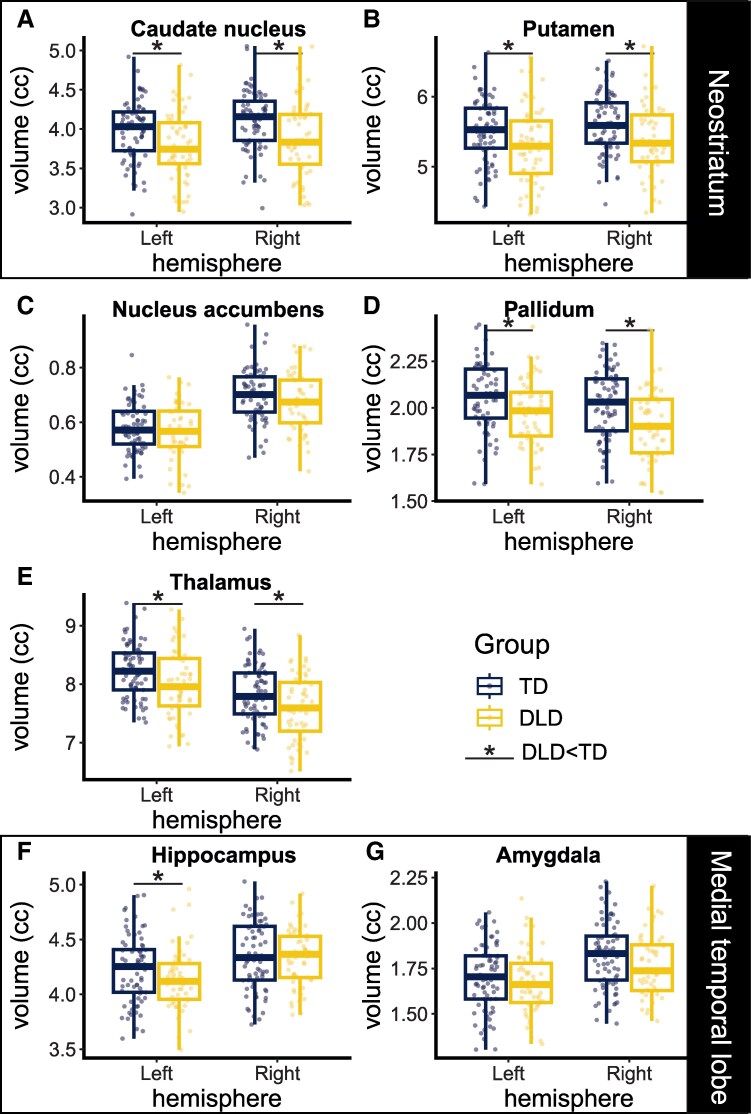
**Subcortical volumes (corrected for ICV) plotted by region, group, and hemisphere.** Each dot is the volume of one participant’s subcortical structure. **A** and **B** show the neostriatum (caudate nucleus, putamen); **C** is the nucleus accumbens; **D** is the pallidum; **E** is the thalamus; **F** and **G** are the medial temporal lobe (hippocampus, amygdala). Full statistical models are shown in Supplementary Table 4. Significance is noted when p_adj_ < 0.05 for a main effect of group or interaction of group × hemisphere in the statistical model that accounts for correlation in structure of the data within participant across hemisphere as well as effects of sex and age (caudate nucleus: β = −3.0, p_adj_ < 0.01, putamen: β = −3.3, p_adj_ < 0.01, pallidum: β = −3.5, p_adj_ < 0.01, thalamus: β = −3.1, p_adj_ < 0.01). In the hippocampus, the main effect of group was significant before correction but not after correction (β = −2.1, *P* < 0.04, p_adj_ = 0.06); the interaction term of group and hemisphere was significant (β = 2.24, *P* < 0.03, p_adj_ < 0.05), indicating that only the left hemisphere was significantly smaller. Caudate nucleus showed an additional interaction effect such that the difference between groups was larger in the right hemisphere (β = −2.8, p_adj_ < 0.02). *P*-values were adjusted to account for multiple comparisons. Volume is indicated in cubic centimetres (cc; mm^3^/1000). Boxplots indicate first quartile, median and third quartile; whiskers show first and third quartile ±1.5*interquartile range. For all plots, *N* = 126–128 participants (outliers removed as discussed in Methods). Abbreviations: DLD = developmental language disorder; ICV = intracranial volume; TD = typically developing.

Graphical representations of the volumetric data are shown in Fig. 2, with group means in Supplementary Table 3 and statistical summary in Supplementary Table 4. The DLD group had smaller volumes relative to the TD group in the caudate nucleus, putamen, globus pallidus, thalamus (significant main effect of group) and left hippocampus (significant interaction of group × hemisphere, see Supplementary Table 4). These structures also differed in size between hemispheres (main effect of hemisphere, see Supplementary Table 4). There was a significant rightwards asymmetry in the caudate nucleus, putamen, nucleus accumbens, hippocampus and amygdala and a significant leftwards asymmetry in the globus pallidus and thalamus. The difference between hemispheres was more pronounced in the TD group than the DLD group for the caudate nucleus volumes (significant interaction between group and hemisphere; rightwards asymmetry was bigger in TD than DLD; group difference in volume was larger in the right hemisphere). Most subcortical structures were larger in male participants (significant effect of sex, see Supplementary Table 4). Age was associated with increased volume in the globus pallidus and hippocampus.

### Microstructural differences

**Figure 3:**
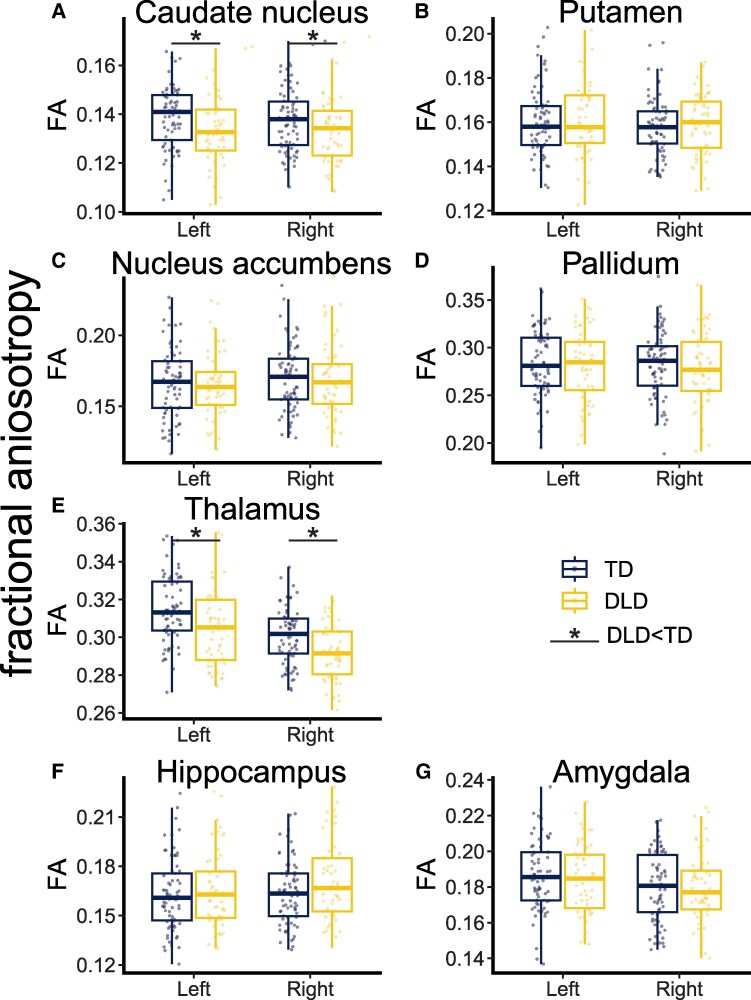
**Microstructure (fractional anisotropy; FA) plotted by region, group and hemisphere.** Each dot is the mean FA of the given subcortical structure for one participant. FA is a ratio (i.e. unitless) and ranges from 0 to 1. **A** and **B** show the neostriatum (caudate nucleus, putamen); **C** is the nucleus accumbens; **D** is the pallidum; **E** is the thalamus; **F** and **G** are the medial temporal lobe (hippocampus, amygdala). Significance is noted when p_adj_ < 0.05 for a main effect of group. in a linear mixed model (caudate nucleus: β = −0.6, p_adj_ = 0.05; thalamus: β = −0.9, p_adj_ < 0.01). Boxplots indicate first quartile, median and third quartile; whiskers show first and third quartile ±1.5*interquartile range. Full statistical tables are in Supplementary Table 5. Each individual data marker is FA from one participant’s subcortical structure. For all plots, *N* = 126–128 participants (outliers removed as discussed in Methods). Abbreviations: DLD = developmental language disorder; FA = fractional anisotropy; TD = typically developing.

To complement the macrostructural differences captured by volumetric analyses, we also evaluated diffusion metrics in subcortical grey matter. FA by group, region and hemisphere is plotted in Fig. 3; plots of MD and full model tables are inSupplementary material (Supplementary Fig. 1; Supplementary Tables 5 and 6). The DLD group had lower FA than TD in the caudate nucleus and thalamus (main effect of group; significant after FDR correction for multiple comparisons). MD did not differ between groups in any structures.

**Figure 4:**
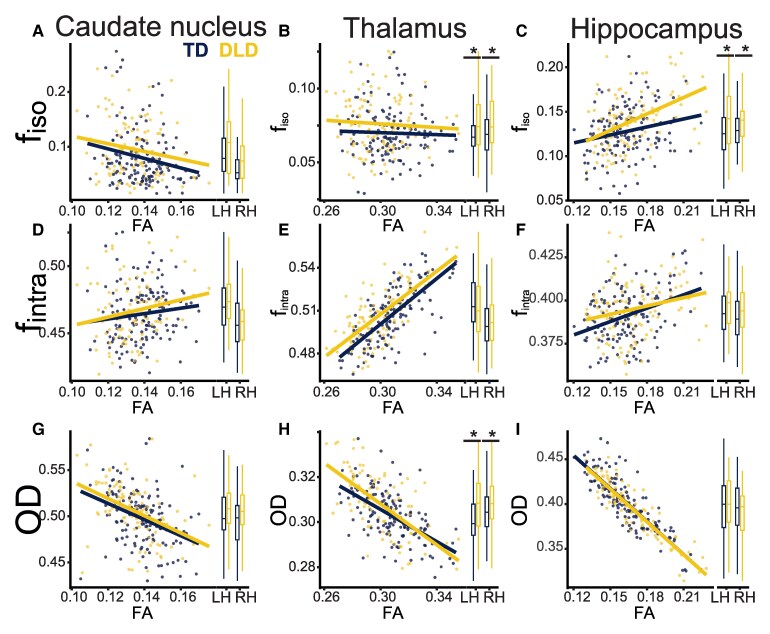
**NODDI contributions to FA and group differences in NODDI parameters (f_iso,_ f_intra_, OD).** Each dot is the microstructural measure of the given subcortical structure of one participant. **A, B and C** show *f*_iso_ in the caudate nucleus, thalamus, and hippocampus, respectively. **D, E and F** show *f*_intra_, and **G, H and I** show OD. Scatterplots show the relationship between NODDI parameter and FA for each participant, with left and right hemispheres plotted together. Boxplots show group differences in NODDI parameters separated by hemisphere. Boxplots indicate first quartile, median and third quartile; whiskers show first and third quartile ±1.5*interquartile range. Significance is denoted by an asterisk and indicates a significant effect of group in a linear mixed model (not corrected for multiple comparisons). Significance shown for: group difference in *f*_iso_ in the thalamus (β = 0.7, *P* < 0.04); group difference in *f*_iso_ in the hippocampus (β = 1.3, *P* < 0.02) and group difference in OD in the thalamus (β = 0.7, *P* < 0.002). Full statistical tables are in Supplementary Tables 7–9. For all plots, *N* = 124–128 participants (outliers removed as discussed in Methods). NODDI parameters and FA are all unitless; FA, *f*_iso_ and *f*_intra_ are proportions (0–1), and OD is also bounded by 0, 1, in which 0 is perfectly aligned straight fibres and 1 is completely isotropic. Abbreviations: DLD = developmental language disorder; ICV = intracranial volume; TD = typically developing; NODDI = Neurite Orientation Dispersion and Density Imaging; FA = fractional anisotropy; OD = orientation dispersion; LH = left hemisphere; RH = right hemisphere.

Multi-compartment diffusion analyses can further explain the microstructural differences underlying FA differences. We used Pearson’s correlations between FA and each NODDI parameter (*f*_iso_, *f*_intra_ and OD) to determine which measures related to FA in the caudate nucleus, thalamus and hippocampus. These relationships, along with group medians in these parameters, are shown in Fig. 4. In the caudate nucleus, there were moderate to weak correlations between FA and OD (ρ = −0.41, *P* < 0.0001), FA and *f*_iso_ (ρ = −0.21, *P* < 0.001) and FA and *f*_intra_ (ρ = 0.14, *P* < 0.03). In the thalamus, there were strong correlations between FA and OD (ρ = −0.63, *P* < 0.0001) and FA and *f*_intra_ (ρ = 0.69, *P* < 0.0001) but no correlation between FA and f_iso_ (ρ = −0.09, *P* < 0.20). In the hippocampus, there was a strong correlation between FA and OD (ρ = −0.88, *P* < 0.0001) and moderate correlations between FA and *f*_intra_ (ρ = 0.31, *P* < 0.0001) and *f*_iso_ (ρ = 0.34, *P* < 0.0001).

We complemented this correlational analysis with linear mixed models on each structure separately (for full results, see Supplementary Tables 7–9). In the caudate nucleus, lower FA was driven primarily by higher OD and *f*_iso_, but group differences in OD and f_iso_ did not reach significance. In the thalamus, lower FA was driven primarily by higher OD and lower f_intra_. We found higher OD (fibres more disperse; β = 0.7, *P* < 0.002) and *f*_iso_ (more cerebrospinal fluid; β = 0.7, *P* < 0.04) in the thalamus of the DLD group. This suggests that at least in the thalamus, FA differences reflect differences in the organization of axons and dendrites as well as the amount of water in extracellular spaces. Even though there was no difference in FA in the hippocampus in DLD, we found higher *f*_iso_ (more cerebrospinal fluid; β = 1.3, *P* < 0.02).

### Multivariate analyses

Multivariate models determined the best brain predictors of language proficiency across the entire cohort as well as within the TD, DLD and HSL groups independently.

#### Model derived from entire cohort

In this study, we attempted to use only brain measures and demographics to predict overall language proficiency. The cohort consisted of 156 participants across TD, DLD and HSL groups. Language proficiency predicted by brain measures was modelled using sex (1 = male, 0 = female), age, handedness and ICV, along with volume, OD, *f*_intra_, *f*_iso_ for seven regions in two hemispheres (another 56 predictors). The best model for predicting language proficiency across the whole cohort is shown below (variables after sex ordered by type of measure):


$$\begin{array}{rl} \mathrm{Language}\;\mathrm{proficiency} & =-0.2\text{–}19\times \mathrm{Sex}\\ & +9.2\times \mathrm{left}\;\mathrm{putamen}\;\mathrm{volume}\;\\ & +7.7\times \mathrm{right}\;\mathrm{caudate}\;\mathrm{volume}\\ & +5.3\times \mathrm{right}\;\mathrm{thalamus}\;\mathrm{volume}\;\\ & \text{–}5.9\times \mathrm{right}\;\mathrm{thalamus}\;f_{\mathrm{iso}}\;\\ & \text{–}5.2\times \mathrm{left}\;\mathrm{hippocampus}\;f_{\mathrm{iso}}\\ & \text{–}0.5\times \mathrm{right}\;\mathrm{caudate}\;f_{\mathrm{iso}}\\ & \text{–}19\times \mathrm{left}\;\mathrm{thalamus}\;\mathrm{OD}\\ & \text{–}5.9\times \mathrm{right}\;\mathrm{caudate}\;\mathrm{OD}\\ & \text{–}2.4\times \mathrm{left}\;\mathrm{accumbens}\;\mathrm{OD}\\ & -0.5\times \mathrm{right}\;\mathrm{accumbens}\;\mathrm{OD}\\ & -0.8\times \mathrm{left}\;\mathrm{hippocampus}\;f_{\mathrm{intra}} \end{array}$$

To summarize, female sex and higher volumes in the left putamen, right caudate nucleus and right thalamus predicted better language proficiency (positive betas). For the microstructural measures (all negative betas), less free water diffusion (f_iso_, contribution from cerebrospinal fluid) in the right thalamus, left hippocampus and right caudate nucleus, less dispersion of neurites (OD) in the left thalamus, right caudate nucleus and left and right nucleus accumbens and less intracellular diffusion (f_intra_) in the left hippocampus predicted higher language proficiency.

**Figure 5:**
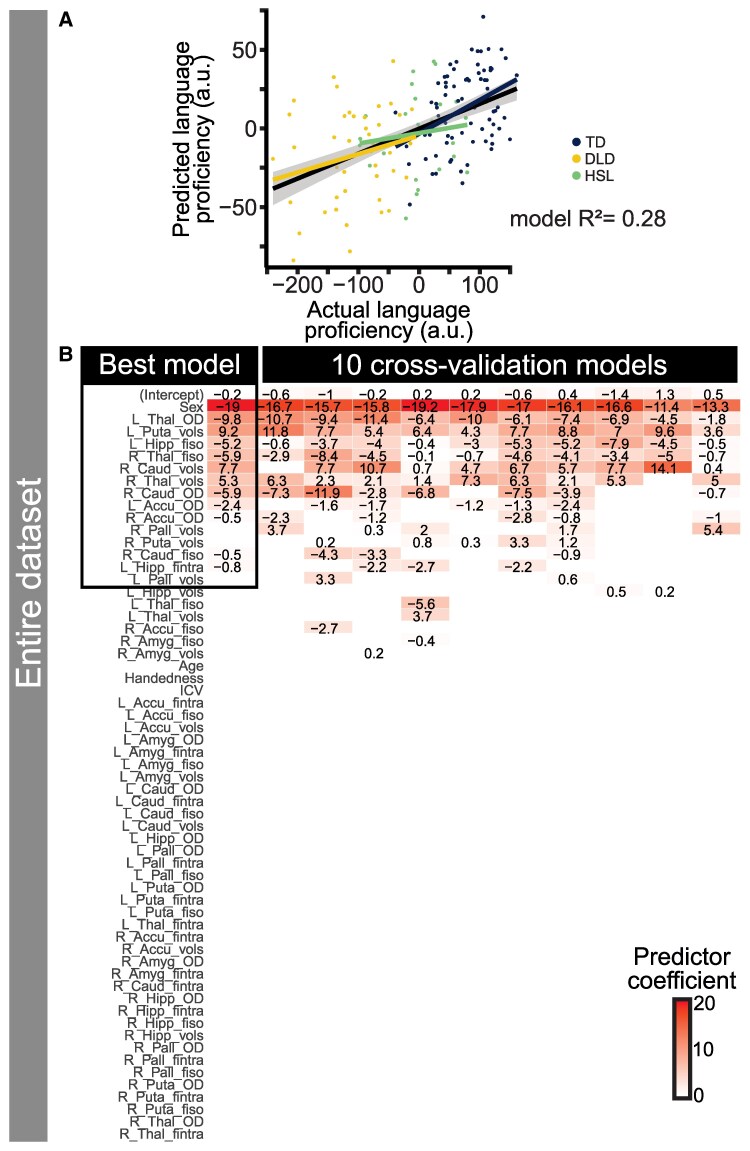
**Results of multivariate analyses across the entire cohort.** (**A**) shows actual language proficiency on *x* axis and predicted language proficiency on *y* axis, where each dot is one participant (*N* = 156). (**B**) shows the predictors selected by machine learning algorithm across all participants in the model (highlighted in a black box) and then each of 10 cross-validation runs with 90% of the data at a time, in order to show the stability of the predictors. Note that the language proficiency range is arbitrary units (a.u.) from −2.5 to 1 but was multiplied by 100 here for readability/interpretability of the terms in the model. Predictors are ordered by the number of times they were selected and then by the absolute value of the beta coefficient (contribution to the model). The model fit is shown within (blue, yellow, green) and across (black) groups. Abbreviations: DLD = developmental language disorder; HSL = history of speech and language concerns; TD = typically developing.

The *R*^2^ value of the model using all the data was 0.28. The predictive value of the model is shown in Fig. 5A, which shows actual language proficiency against predicted language proficiency. Many variables were reliably chosen across 10-fold cross-validation, with the best model (lowest error) for each fold shown in Fig. 5B. All cross-validation models included sex (males < females), left putamen volume (higher volumes, higher language proficiency), OD in the left thalamus (higher OD, lower language proficiency) and isotropic diffusion in the right thalamus and left hippocampus (higher diffusion, lower language proficiency). Most models also included the volume of the right caudate nucleus and left thalamus. The regions identified by the modelling as significant predictors of language proficiency are largely those that were (individually) significantly different between the groups, which is to be expected given that the language factor also differs between groups. Supplementary Fig. 2 shows scatterplots of select subcortical volumes against language proficiency to demonstrate the predictive value of individual predictors.

#### Model derived from TD group

**Figure 6:**
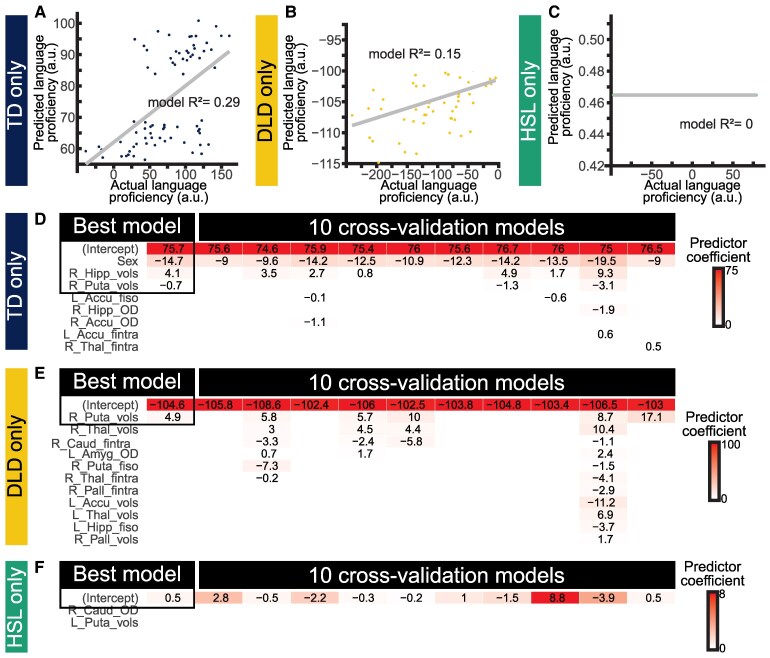
**Results of multivariate analyses within each group.** (**A–C**) show actual language proficiency on *x* axis and predicted language proficiency on *y* axis for TD (*N* = 74), DLD (*N* = 54) and HSL (*N* = 28) groups, respectively; each dot is one participant. (**D–F**) show the predictors selected by the machine learning algorithm across all participants in the model (highlighted in a black box) and then each of 10 cross-validation runs with 90% of the data at a time, in order to show the stability of the predictors. See legend to Fig. 5 for details. Note that hippocampus measures are selected for the entire dataset and TD model but not for DLD or HSL models. Abbreviations: DLD = developmental language disorder; HSL = history of speech and language concerns; TD = typically developing.

To explore the brain–behaviour relationships within the group, the model selection process was completed on each group individually. Separate models for the TD and DLD groups could, for example, reveal relationships that are only related to one group, which would indicate possible differences in learning strategies or mechanisms. Results for the TD group are shown in Fig. 6A and D. The best TD model was


$$\begin{array}{rl} \mathrm{Language}\;\mathrm{proficiency}(\mathrm{TD}) & =75.7\text{–}14.7\times \mathrm{sex}\\ & +4.1\times \;\mathrm{right}\;\mathrm{hippocampus}\;\mathrm{volume}\\ & \text{–}0.7\times \mathrm{right}\;\mathrm{putamen}\;\mathrm{volume} \end{array}$$

The TD model had sex as a consistent factor, and interestingly, the volume of the right hippocampus positively contributing to language proficiency was selected in 7/10 models. The *R²* value of the model using all the data was 0.29, a slightly better fit than the models including DLD and HSL cohorts.

#### Model derived from DLD group

When evaluating the DLD group alone, the best fit model was language proficiency = −104.6 × sex + 4.9 × right putamen volume, for a final *R*^2^ = 0.15 (Fig. 6B and E). Plots of language proficiency within the DLD group versus subcortical structure volume are shown in Supplementary Fig. 2.

#### Model derived from HSL group

When evaluating the HSL group alone, the machine learning algorithm routinely removed all predictors, indicating that none were stable predictors of language proficiency (Fig. 6C and F), and resulting in an *R*^2^ of 0. These analyses indicate that subcortical volumes and microstructure are not predictive of language proficiency within the HSL group.

#### Effect of sample size

Both DLD and HSL groups are smaller than the TD group (TD: 74; DLD: 54; HSL: 28) and therefore offer fewer exemplars for learning the data. To ensure that sample size alone was not affecting the ability of the models to predict language proficiency, we bootstrapped the TD data to select 54 participants randomly, 100 times and recalculated the TD-only model on these smaller cohorts. In all cases, some parameters were kept from the LASSO process, leading to a wide variety of explanatory power (*R*^2^ = 0.06–0.79, mean: 0.28). Seventy-seven of 100 samples used brain predictors, the most common of which was the volume of the right hippocampus (62/100), followed by the volume of the right putamen (31/100). Even on only 28 participants (HSL group size), of the 100 models run, the TD models retained parameters in 63 runs, with explanatory power of 0.00–0.99 (mean: 0.28).

#### Predicting memory instead of language

To help put these results into perspective, we also used identical methods to predict verbal memory proficiency using the same macro- and microstructural measures across the entire cohort. Interestingly, even though the language and memory factors are correlated (*r* = 0.687; see^20^ for a plot of the relationship), the subcortical model to predict memory proficiency was much less successful: *R*^2^ = 0.06 compared to *R*^2^ = 0.28 for the language model. Plots of the fit and machine learning results are shown in Supplementary Fig. 3.

## Discussion

In this study, we scanned and analysed MRI data from 156 children and adolescents, 74 who had typical development (TD), 54 who met criteria for DLD and 28 with a history of speech and language difficulties but did not meet criteria for DLD (HSL). Nearly all participants in the DLD group performed poorly on tests of receptive and expressive sentence processing (grammar) with more variability in performance on tests of narrative and single words (vocabulary) (see Fig. 1). In addition to these language difficulties, the DLD group also showed lower performance compared with the TD group across a range of tasks including memory, non-verbal reasoning, reading and motor skills (see Table 1).^20,22^ The performance of the HSL group was more varied relative to the other two groups. Masks of subcortical structures were derived from FreeSurfer and used to calculate volume and diffusion measures in the regions including the neostriatum (caudate nucleus, putamen) and medial temporal lobe (hippocampus, amygdala). Compared with the TD group, we found that the DLD group had smaller volumes across multiple subcortical structures bilaterally: caudate nucleus, putamen, globus pallidus, thalamus and hippocampus. Microstructural analyses using diffusion imaging data found lower FA bilaterally in the caudate nucleus and thalamus in the DLD group relative to the TD group; there were no differences in MD in any structures. NODDI imaging revealed that the DLD group compared with the TD group had non-specific changes in the caudate nucleus but showed both higher levels of free water diffusion (increased *f*_iso_; which corresponds to the amount of CSF in a voxel) and less organized neurites (increased OD; which corresponds to the dispersion of orientation of axons and dendrites in a voxel) in the thalamus. Using data from the whole cohort, including the HSL group, and multivariate machine learning, we explored the relationships between summary measures of language and memory proficiency and the brain measures. We first discuss our findings and then provide interpretations of the work that indicate areas of future research.

### Predictions from the PCDH

The predictions from the PCDH explored here were that the neostriatum, which contributes to procedural learning, would be smaller in DLD and that medial temporal lobe structures, associated with declarative learning, would be spared, potentially allowing them to compensate for the impairment in procedural learning.

Our findings confirmed the first prediction. We replicated the most consistent finding from previous macrostructural studies of DLD, namely that the anterior neostriatum is affected in DLD.^8^ In our large cohort, the volumes of the caudate nucleus and putamen were lower in DLD than in TD. However, the second prediction, that the medial temporal lobe structures involved in declarative learning were spared in DLD, was not upheld. Specifically, our data indicated that the left hippocampus is *smaller* in DLD. Furthermore, the pallidum and the thalamus were also found to be smaller in DLD. The PCDH offered no predictions about the role of the pallidum or thalamus in the language learning difficulties experienced by individuals with DLD, though they are part of the basal ganglia circuitry that supports procedural learning, receiving inputs from the striatum and sending outputs to the cortex.

A related prediction from the PCDH is that the unaffected medial temporal lobe system involved in declarative learning could compensate for the procedural learning deficit in DLD. It is unclear whether this potential compensatory activity would have consequences for the medial temporal lobe structures in terms of their macro- or microstructure. We did not directly test this prediction, but our measures of episodic memory—the word list learning subtest of the Children’s Memory scale—indicated lower average scores for DLD relative to TD on immediate and delayed recall and delayed recognition. Based on this behavioural difference and smaller volume in DLD, we think it is unlikely that the hippocampus plays a compensatory role in language learning in this group.

Several studies point to a role for the hippocampus in language learning. For instance, one theoretical account proposes that the hippocampus supports initial, fast word-referent associative learning that is then transferred to the cortex via consolidation.^67^ Another emphasizes how the pattern of hippocampal functional connectivity predicted performance on an artificial grammar learning task.^68^ Hippocampal volume increases with intensive language learning^69^ and is maintained in bilinguals who continue to use multiple languages.^70^ Recent models of hippocampal function suggest that the hippocampus may be involved with the creative and flexible use of language, which is also reduced in those with acquired hippocampus damage.^71^ In young children, the volume of the right hippocampus predicted statistical learning ability.^72^ In children and adults, superior performance on tests of statistical learning and associative inference, which required encoding associations across multiple events, was related to a smaller volume of the hippocampal head, bilaterally.^73^ Accordingly, our finding of a smaller hippocampal volume in DLD is consistent with their language learning impairment.

A smaller volume of the hippocampus is not specific to DLD nor to language learning impairment, however. Abnormal hippocampal structure is found in association with other neurodevelopmental conditions, including schizophrenia.^74^ For example, smaller hippocampal volume is linked to impairments in social interactions in autism spectrum disorder^75^ and to subtypes of attention-deficit hyperactivity disorder in children and adolescents.^76^ Taken together, these findings may reflect a specific vulnerability of the hippocampus during development.

Finally, it is worth considering our group of participants with a history of speech and language difficulties, who did not meet criteria for DLD at the time of testing. In all cases, they were (behaviourally) in between the performance of the DLD group and the TD group. Since they all had a history of concerns or diagnoses, this HSL group may include participants who are ‘recovered’ or have subthreshold DLD and could therefore be considered to have successfully compensated for their language learning difficulties. However, the HSL group also scored lower than the TD group on our test of hippocampal function, namely the word list learning subtest of the Children’s Memory Scale (see Table 1). It is unlikely, therefore, that they have used episodic memory and the hippocampus to compensate.

### Myelin differences across *in vivo* methods

We previously described quantitative microstructural tissue differences in a subset of this cohort who had usable multiparameter map scans.^30^ In that whole brain voxel-wise analysis, two measures related to myelin density were significantly lower in DLD: (i) the whole brain average of longitudinal relaxation rate (R1); (ii) magnetization transfer saturation (MTsat) in the dorsal striatum and thalamus bilaterally, along with several left hemisphere cortical areas classically involved in language. Considering our current findings together with this previous work, we conclude that the neostriatum (dorsal striatum) is both smaller in volume and that the tissue that is there has fewer processes and less myelin. To investigate this further, we used diffusion imaging data to probe the microstructure of these subcortical grey matter structures more closely.

In the current study, we found lower FA in the subcortical grey matter of the caudate nucleus and thalamus bilaterally. Since lower FA can reflect lower myelin content and also be caused by a variety of microstructural differences, including fewer neurites (axons, dendrites), smaller axon diameter, more water (e.g. oedema) or less spatial coherence of the neurites (i.e. less ‘well-organized’), we used NODDI (see methods) to understand whether the FA measure was capturing the reduced myelin content seen in the previous study of a subset of this sample or whether there are additional differences in the fibre microstructure, such as their organization or packing density.

In the thalamus, there were group differences in both free-water diffusion (isotropic, *f*_iso_) and OD, suggesting that the lower FA seen in DLD is due to both more cerebrospinal fluid in the tissue and less organized processes (which include myelinated axons). This finding is consistent with the multiparameter mapping results showing reduced myelin content in the thalamus in DLD and a global reduction in neurites reflected by the lower whole-brain R1.^30^ We found that FA in the caudate nucleus was moderately correlated with both OD and free-water diffusion, but there were no significant group differences in these parameters individually. We interpret these findings cautiously since replication and further study are warranted. Overall, a future longitudinal study is needed to determine how these differences arise and change over time.

It is worth noting that although hippocampal volume was smaller in DLD, there was no difference in FA or MD. Of the microstructural properties explored, only free-water diffusion (*f*_iso_) differed in the DLD group, consistent with more cerebrospinal fluid in the tissue.

### Grey matter volume and myelin in subcortical grey matter

White matter is primarily composed of myelinated axons. However, along with neuronal cell bodies and dendrites, grey matter also contains myelinated and unmyelinated axons.^77^ Myelin within grey matter is rarely studied, and when it is, it is cortical grey matter that is considered. Interestingly, the myelination within grey matter continues into and beyond adolescence, depending on the region. Grey matter *volume* measurements across the lifespan are generally inverted-U shaped, increasing in infancy and reaching a peak, then declining into late adulthood. In neurotypical children, caudate nucleus volumes decrease from the ages 7–25 years,^78^ although hippocampus and globus pallidus volumes continue to rise in the age range of the current study (10–15 years) before plateauing and decreasing after age 20. In the hippocampus, then, we could consider the lower volume as an indication that the DLD group is maturationally behind the TD group. In the caudate nucleus, however, less mature structures would be *larger* in this age range.^78^ Furthermore, grey matter volume reduction with age through adolescence into adulthood could be related to either increasing myelination, or to synaptic pruning, cell loss and shrinkage.^77^ However, the DLD cohort had reduced FA in the caudate nucleus along with reduced volumes. Thus, in the caudate nucleus, something other than a delayed maturation account (which would result in larger volumes and less myelin in the DLD group) is needed to explain the pattern of results; longitudinal studies may be able to indicate what is different between groups and what is changing within participants over this age range, including differences in the number of cell bodies and neurites.

### Beyond univariate analyses: predicting language ability from multiple measures

In our univariate analyses, subcortical nuclei were considered individually, and their differences were assessed categorically by group. Our multivariate analyses probed whether combining the effects across different structure metrics would provide power for predicting a measure of language proficiency rather than purely group differences. We included a group of children with a history of language difficulties but who did not meet criteria for DLD at the time of testing. Behaviourally, this group had scores on language and memory factors that typically fell between those of the DLD and TD groups (see Table 1). Combining data across groups allowed us to explore the full range of language learning abilities in our cohort. The model revealed that sex, macrostructural measures of the left putamen, right caudate and right thalamus, along with measures of the microstructure of the thalamus, nucleus accumbens, right caudate and left hippocampus were predictive of language proficiency across the cohort. This model, which included only measures of brain structure (in addition to sex, age and handedness), explained 28% of the variance in language proficiency. Adding other potential predictors, for example, measures of genetic and environmental risk factors, cortical structure and brain function, would potentially improve the model fit. To put these findings in context, we also produced a model attempting to predict verbal memory performance from the same subcortical data. That model only explained 6% of the behavioural variance, using only sex. This demonstrates that language proficiency is actually somewhat well-represented within subcortical structures, whereas verbal memory proficiency is not. This could indicate that these memory tests, which rely largely on long-term memory, depend on cortical regions in the medial temporal lobe, or more generally that subcortical differences do not account well for the variance in memory performance.

Interestingly, and once again contradictory to the predictions from the PCDH, we found that although hippocampus measures were selected as a factor predicting language performance in the whole cohort (Fig. 5B), this effect appears to be limited to the TD group (Fig. 6D) and was not evident in the DLD or HSL groups. The failure of the models to predict language proficiency in the DLD and HSL groups is not due to their smaller sample size. Another explanation is needed, therefore.

Three explanations seem plausible separately or in conjunction as we are attempting to relate patterns of brain structure to behaviour. (1) While the DLD group does not appear significantly more variable on each structure size and microstructure (Fig. 2, Fig. 4), the multivariate analyses may be suggesting that the *pattern* of structure volume and microstructure itself is more variable within the DLD group. (2) The DLD group has more variance on the language proficiency measure, so it could be that this behavioural variability is harder to predict. (3) It could be that the relationship between language and brain structure is more variable in the DLD group. These explanations are all plausible, particularly as we know that many different brain structural differences could lead to different behaviour and vice versa. In addition, each individual may have had different behavioural and neural processes attempting to compensate for language differences throughout development. The children with typical language and (putatively) typical brain development may have more consistent brain structures and more consistent behaviour–brain relationships. We would highlight that it is interesting that these limited data about subcortical structures are able to predict behaviour in any capacity, particularly given the relevance of the cortex for language processing.

### Thalamus: underexplored in DLD?

The thalamus plays a key role as a relay station for basal ganglia and cerebellar nuclei output to the cortex and has reciprocal cortical projections. Reports in children and adults with typical language have also implicated the thalamus in a variety of language functions,^79,80^ including those that are impaired in DLD like non-word repetition,^81^ semantics and syntax. DLD has also been associated with slower processing speed across tasks,^82^ which could be related to the gating and coordination functions of the thalamus.^83,84^ It would thus be unsurprising if the thalamus were affected in DLD. However, very few differences in the thalamus were reported in a meta-analysis (1.1% likelihood of structural differences, 0% likelihood of functional MRI differences),^8^ even though one previous study using diffusion measures^13^ and the current study found macro- and microstructural differences in the thalamus in DLD. A PET study in children with DLD showed atypical glucose metabolism largely in the thalamus.^85^ A well-powered structural (VBM) study of dyslexia across languages found *only* differences in the left thalamus^86^; a recent interesting study of thalamic function and microstructure also highlighted differences in dyslexia.^87^ Future study may elucidate the role of the thalamus in DLD and identify the specific nuclei involved. Longitudinal studies may also be able to pinpoint whether differences in the thalamus are primary in DLD, a consequence of other structural differences leading to atypical thalamic organization, or a consequence of atypical language experience.

## Conclusion

Our study in a large cohort of children (*N* = 156: 54 DLD, 74 TD, 28 HSL) revealed differences across several subcortical regions, including those that the PCDH predicted were unaffected. We propose that while neostriatal deficits are present in DLD, differences are not confined to this region. Instead, theoretical frameworks are needed that account for neural and behavioural deficits across many domains and perhaps across the brain. Longitudinal or genetic studies may reveal that neostriatal differences are indeed primary to the disorder, but that cannot be determined with the methods currently in use. Collaborative research (and funding) is needed to collect and analyse high-quality larger datasets capable of capturing both small effects and the range of neural and behavioural heterogeneity characteristic of DLD. The PCDH should be updated to reflect a potential role for the hippocampus in the language learning difficulties experienced by individuals with DLD and to remove the suggestion that the hippocampus could compensate for these difficulties. Even so, our findings do not rule out a potential compensatory role for other medial temporal structures, for example, the amygdala and temporal lobe neocortex.

## Supplementary Material

fcaf493_Supplementary_Data

## Data Availability

Anonymized behavioural scores, brain measures and scripts supporting the findings of this study are openly available on the Open Science Framework (https://osf.io/wmkrq/).
