# Supplementary material for: Subcortical correlates of developmental language disorder: more than the neostriatum
Source: Brain Commun. 2025 Dec 17;8(1):fcaf493. doi: 10.1093/braincomms/fcaf493 (PMC12809564; doi:10.1093/braincomms/fcaf493)
Supplement: fcaf493_Supplementary_Data [file fcaf493_supplementary_data.docx]

Supplemental Table 1. Current literature in subcortical differences in DLD. Note that segmentation methods return results in mm^3^ or voxels and represent volume differences. VBM results represent differences in the amount of grey matter in a brain area. Studies ordered by method and date. (abbreviations: ICV: intracranial volume; CN: caudate nucleus; Put: putamen; NA: nucleus accumbens; GP: globus pallidus; Thal: thalamus; Hipp: hippocampus; Amyg: amygdala). > indicates DLD>TD; < is DLD<TD

|  | N | | Age (years) | Method | ICV | CN | | Put | | NA | | GP | | Thal | | Hipp | | Amyg | |
| --- | --- | --- | --- | --- | --- | --- | --- | --- | --- | --- | --- | --- | --- | --- | --- | --- | --- | --- | --- |
|  | DLD | TD |  |  |  | L | R | L | R | L | R | L | R | L | R | L | R | L | R |
| Jernigan et al. (1991) | 20^a^ | 16 | 8–10 | Segmentation |  |  | <^b^ |  |  |  |  |  |  |  |  |  |  |  |  |
| Herbert et al. (2003) | 24 | 30 | 5–11 | Segmentation | > | <^c^ | |  |  |  |  |  |  |  |  |  | | | |
| Lee et al. (2013)^d^ | 12 | 12 | M=22 | Segmentation | < | < | < |  |  |  |  | < |  | < | < |  |  | n/a | n/a |
|  |  |  |  |  |  |  |  | > | > | > | > |  | > |  |  | > | > | n/a | n/a |
| Soriano-Mas et al. (2009) | 36 | 36 | 5–17 | VBM |  |  | >^e^ |  |  |  |  |  |  |  |  |  |  |  |  |
| Badcock et al. (2012) | 10 | 16 | 8–17 | VBM | ns^f^ |  | < |  |  |  |  |  |  |  |  |  |  |  |  |
| Girbau-Massana et al. (2014) | 10 | 14 | M=10;1 | VBM |  |  |  |  |  |  |  |  |  |  |  |  |  |  |  |
| Pigdon et al. (2019) | 17 | 40 | 9–11 | VBM |  |  |  |  | >^g^ |  |  |  |  |  |  |  |  |  |  |

n/a: not assessed

^a^ participants were language- *and* learning-impaired, although all but 1 would meet DLD criteria (non-verbal IQ>70)

^b^ results significant only if left-handed participants were removed

^c^ assessed left and right volumes together; only different when corrected for larger ICV.

^d^ ran analyses with and without correcting for ICV, which differed between groups. Note also that this cohort is adult and had an atypical gender balance for this population (twice as many women as men). Top row is volumes uncorrected for ICV. Bottom row is volumes corrected for ICV.

^e^ significant when only younger children were considered

^f^ not reported in paper but confirmed by senior author

^g^ only uncorrected *p* is significant

Supplemental Table 2. Linear model results probing group differences in intracranial volume.

|  | **ICVcc** | | |
| --- | --- | --- | --- |
| *Predictors* | *Estimates* | *Statistic* | *p* |
| (Intercept) | 1493.72 | 9.59 | **<0.001** |
| Group [DLD] | -21.30 | -0.08 | 0.934 |
| Age | -18.65 | -1.52 | 0.132 |
| Sex [Male] | -417.55 | -2.00 | **0.048** |
| Group [DLD] * Age | -5.81 | -0.29 | 0.774 |
| Group [DLD] * Sex [Male] | 242.61 | 0.76 | 0.452 |
| Age * Sex [Male] | 44.62 | 2.71 | **0.008** |
| (Group [DLD] * Age) * Sex [Male] | -13.09 | -0.52 | 0.607 |
| Observations | 128 | | |
| R^2^ / R^2^ adjusted | 0.380 / 0.344 | | |

**Supplemental Table 3. Mean values for each of the** measures in each region, hemisphere, and group. Values shown as mean (standard deviation). Region volumes are corrected for ICV. MD values should be multiplied by 10^-4^. Individual data points greater than 2.5 SDs above or below the mean of the TD+DLD groups have been removed.

|  |  | **TD** | | **DLD** | | **HSL** | |
| --- | --- | --- | --- | --- | --- | --- | --- |
|  |  | **Left** | **Right** | **Left** | **Right** | **Left** | **Right** |
| **Volume (cc)** | ICV | 1339.6 (136.5) | | 1323.6 (164.6) | | 1409.5 (184.1) | |
|  | Caudate nucleus | 4.0 (0.4) | 4.1 (0.4) | 3.8 (0.4) | 3.9 (0.5) | 3.9 (0.5) | 4.0 (0.5) |
|  | Putamen | 5.5 (0.5) | 5.6 (0.4) | 5.3 (0.5) | 5.4 (0.5) | 5.6 (0.4) | 5.6 (0.4) |
|  | Accumbens | 0.6 (0.1) | 0.7 (0.1) | 0.6 (0.1) | 0.7 (0.1) | 0.6 (0.1) | 0.7 (0.1) |
|  | Pallidum | 2.1 (0.2) | 2.0 (0.2) | 2.0 (0.2) | 1.9 (0.2) | 2.1 (0.2) | 2.0 (0.2) |
|  | Thalamus | 8.2 (0.5) | 7.8 (0.5) | 8.0 (0.6) | 7.6 (0.6) | 8.2 (0.5) | 7.7 (0.5) |
|  | Hippocampus | 4.2 (0.3) | 4.3 (0.3) | 4.1 (0.3) | 4.3 (0.3) | 4.2 (0.3) | 4.4 (0.4) |
|  | Amygdala | 1.7 (0.2) | 1.8 (0.2) | 1.7 (0.2) | 1.8 (0.2) | 1.7 (0.1) | 1.8 (0.1) |
|  |  |  |  |  |  |  |  |
| **FA** | Caudate nucleus | 0.14 (0.01) | 0.14 (0.01) | 0.13 (0.01) | 0.13 (0.01) | 0.14 (0.01) | 0.14 (0.01) |
|  | Putamen | 0.16 (0.01) | 0.16 (0.01) | 0.16 (0.02) | 0.16 (0.01) | 0.16 (0.02) | 0.16 (0.01) |
|  | Accumbens | 0.17 (0.02) | 0.17 (0.02) | 0.17 (0.02) | 0.17 (0.02) | 0.17 (0.03) | 0.17 (0.02) |
|  | Pallidum | 0.28 (0.03) | 0.28 (0.03) | 0.28 (0.04) | 0.28 (0.04) | 0.29 (0.04) | 0.29 (0.04) |
|  | Thalamus | 0.31 (0.02) | 0.30 (0.01) | 0.31 (0.02) | 0.29 (0.01) | 0.32 (0.02) | 0.30 (0.02) |
|  | Hippocampus | 0.16 (0.02) | 0.16 (0.02) | 0.17 (0.02) | 0.17 (0.02) | 0.17 (0.02) | 0.17 (0.02) |
|  | Amygdala | 0.19 (0.02) | 0.18 (0.02) | 0.18 (0.02) | 0.18 (0.02) | 0.19 (0.02) | 0.19 (0.02) |
|  |  |  |  |  |  |  |  |
| **MD (x 10^-4^)** | Caudate nucleus | 8.0 (0.7) | 7.8 (0.4) | 8.0 (0.6) | 7.9 (0.5) | 8.1 (0.7) | 7.9 (0.5) |
|  | Putamen | 7.3 (0.1) | 7.3 (0.1) | 7.3 (0.2) | 7.4 (0.2) | 7.3 (0.2) | 7.3 (0.2) |
|  | Accumbens | 8.3 (0.3) | 8.5 (0.3) | 8.3 (0.3) | 8.4 (0.3) | 8.3 (0.4) | 8.4 (0.3) |
|  | Pallidum | 7.2 (0.2) | 7.3 (0.2) | 7.2 (0.2) | 7.2 (0.2) | 7.2 (0.3) | 7.2 (0.3) |
|  | Thalamus | 7.6 (0.2) | 7.7 (0.2) | 7.6 (0.2) | 7.8 (0.2) | 7.6 (0.2) | 7.7 (0.1) |
|  | Hippocampus | 10.0 (0.6) | 9.9 (0.5) | 10.0 (0.6) | 10.1 (0.6) | 10.1 (0.6) | 10.0 (0.6) |
|  | Amygdala | 8.4 (0.4) | 8.3 (0.3) | 8.4 (0.3) | 8.3 (0.3) | 8.5 (0.4) | 8.4 (0.3) |
|  |  |  |  |  |  |  |  |
| **f_iso_** | Caudate nucleus | 0.10 (0.07) | 0.07 (0.05) | 0.12 (0.10) | 0.09 (0.06) | 0.11 (0.06) | 0.09 (0.05) |
|  | Putamen | 0.02 (0.00) | 0.02 (0.00) | 0.02 (0.00) | 0.02 (0.00) | 0.02 (0.00) | 0.02 (0.01) |
|  | Accumbens | 0.03 (0.01) | 0.03 (0.02) | 0.04 (0.03) | 0.04 (0.02) | 0.04 (0.02) | 0.04 (0.03) |
|  | Pallidum | 0.06 (0.01) | 0.06 (0.01) | 0.06 (0.01) | 0.06 (0.01) | 0.06 (0.01) | 0.06 (0.01) |
|  | Thalamus | 0.07 (0.02) | 0.07 (0.02) | 0.08 (0.02) | 0.08 (0.02) | 0.08 (0.02) | 0.08 (0.03) |
|  | Hippocampus | 0.13 (0.03) | 0.13 (0.03) | 0.14 (0.04) | 0.14 (0.04) | 0.13 (0.03) | 0.13 (0.04) |
|  | Amygdala | 0.06 (0.02) | 0.06 (0.03) | 0.06 (0.02) | 0.07 (0.02) | 0.06 (0.02) | 0.07 (0.03) |
|  |  |  |  |  |  |  |  |
| **f_intra_** | Caudate nucleus | 0.47 (0.02) | 0.46 (0.02) | 0.48 (0.03) | 0.46 (0.02) | 0.47 (0.02) | 0.46 (0.02) |
|  | Putamen | 0.50 (0.01) | 0.49 (0.01) | 0.50 (0.02) | 0.49 (0.02) | 0.50 (0.02) | 0.49 (0.02) |
|  | Accumbens | 0.41 (0.02) | 0.39 (0.02) | 0.41 (0.02) | 0.40 (0.02) | 0.42 (0.02) | 0.40 (0.02) |
|  | Pallidum | 0.57 (0.03) | 0.57 (0.03) | 0.57 (0.04) | 0.57 (0.03) | 0.57 (0.03) | 0.58 (0.04) |
|  | Thalamus | 0.51 (0.02) | 0.50 (0.02) | 0.51 (0.02) | 0.50 (0.02) | 0.51 (0.02) | 0.50 (0.02) |
|  | Hippocampus | 0.39 (0.02) | 0.39 (0.02) | 0.40 (0.02) | 0.39 (0.02) | 0.39 (0.02) | 0.39 (0.02) |
|  | Amygdala | 0.42 (0.02) | 0.41 (0.02) | 0.41 (0.02) | 0.41 (0.02) | 0.41 (0.02) | 0.41 (0.02) |
|  |  |  |  |  |  |  |  |
| **OD** | Caudate nucleus | 0.50 (0.03) | 0.50 (0.03) | 0.50 (0.04) | 0.50 (0.04) | 0.50 (0.04) | 0.50 (0.05) |
|  | Putamen | 0.50 (0.03) | 0.50 (0.03) | 0.50 (0.03) | 0.50 (0.02) | 0.50 (0.03) | 0.50 (0.03) |
|  | Accumbens | 0.46 (0.04) | 0.44 (0.03) | 0.47 (0.04) | 0.45 (0.04) | 0.47 (0.04) | 0.44 (0.05) |
|  | Pallidum | 0.39 (0.04) | 0.38 (0.03) | 0.40 (0.04) | 0.39 (0.04) | 0.39 (0.04) | 0.38 (0.04) |
|  | Thalamus | 0.30 (0.01) | 0.30 (0.01) | 0.31 (0.01) | 0.31 (0.02) | 0.30 (0.01) | 0.30 (0.01) |
|  | Hippocampus | 0.40 (0.04) | 0.40 (0.03) | 0.40 (0.03) | 0.39 (0.04) | 0.39 (0.04) | 0.39 (0.04) |
|  | Amygdala | 0.41 (0.03) | 0.42 (0.03) | 0.41 (0.03) | 0.42 (0.03) | 0.40 (0.03) | 0.41 (0.03) |

**Supplemental Table 4. Group differences in subcortical volumes.** Statistical results of linear mixed models on subcortical volumes, showing parameter estimates in mm^3^ and standardized beta coefficients as the statistic (also called *t* by lme4). *p*-vals are adjusted for multiple comparisons (Benjamini-Hochberg correction). Grey boxes are factors that were marginally significant before correction and non-significant post correction. Green highlights significant effect of group (main or interaction).

|  | **Caudate nucleus volume** | | | **Putamen volume** | | | **Nucleus accumbens volume** | | | **Pallidum volume** | | | **Thalamus volume** | | | **Hippocampus volume** | | | **Amygdala volume** | | |
| --- | --- | --- | --- | --- | --- | --- | --- | --- | --- | --- | --- | --- | --- | --- | --- | --- | --- | --- | --- | --- | --- |
| *Predictors* | *Estimates* | *Statistic* | *p-adj* | *Estimates* | *Statistic* | *p-adj* | *Estimates* | *Statistic* | *p-adj* | *Estimates* | *Statistic* | *p-adj* | *Estimates* | *Statistic* | *p-adj* | *Estimates* | *Statistic* | *p-adj* | *Estimates* | *Statistic* | *p-adj* |
| (Intercept) | 4696.74 | 9.88 | **<0.001** | 6878.12 | 12.66 | **<0.001** | 739.83 | 7.21 | **<0.001** | 2329.41 | 12.36 | **<0.001** | 8848.53 | 15.31 | **<0.001** | 4191.58 | 13.13 | **<0.001** | 2175.89 | 11.74 | **<0.001** |
| hemisphere [Right] | 151.11 | 8.57 | **<0.001** | 93.06 | 3.51 | **0.003** | 115.68 | 11.65 | **<0.001** | -51.16 | -2.98 | **0.009** | -392.11 | -10.48 | **<0.001** | 119.38 | 4.74 | **<0.001** | 117.68 | 7.09 | **<0.001** |
| Group [DLD] | -224.13 | -3.00 | **0.009** | -281.93 | -3.27 | **0.005** | -24.82 | -1.40 | 0.228 | -110.42 | -3.46 | **0.003** | -287.18 | -3.07 | **0.007** | -111.89 | -2.12 | 0.061 | -61.15 | -1.95 | 0.087 |
| Age | 6.26 | 0.31 | 0.796 | -5.67 | -0.25 | 0.833 | -4.72 | -1.10 | 0.346 | 18.91 | 2.41 | **0.034** | 44.77 | 1.85 | 0.106 | 32.04 | 2.40 | **0.034** | 5.71 | 0.74 | 0.539 |
| Sex [Female] | -234.95 | -2.61 | **0.022** | -338.47 | -3.30 | 0.004 | -32.93 | -1.71 | 0.137 | -115.07 | -3.24 | **0.005** | -308.02 | -2.82 | **0.013** | -101.30 | -1.68 | 0.140 | -142.17 | -4.06 | **<0.001** |
| Handedness [Left] | -152.65 | -1.52 | 0.189 | -130.64 | -1.14 | 0.335 | -49.96 | -2.25 | **0.047** | -27.40 | -0.69 | 0.562 | -23.53 | -0.19 | 0.863 | -9.87 | -0.15 | 0.884 | -47.64 | -1.22 | 0.302 |
| ICVcc | -0.50 | -1.75 | 0.128 | -0.84 | -2.58 | **0.023** | -0.05 | -0.89 | 0.458 | -0.33 | -2.96 | **0.009** | -0.78 | -2.24 | 0.047 | -0.24 | -1.23 | 0.300 | -0.35 | -3.17 | **0.006** |
| hemisphere [Right] × Group [DLD] | -76.32 | -2.81 | **0.013** | -19.13 | -0.47 | 0.692 | -12.00 | -0.78 | 0.522 | -14.80 | -0.56 | 0.649 | 30.86 | 0.53 | 0.654 | 87.61 | 2.24 | **0.047** | -26.89 | -1.06 | 0.363 |
| **Random Effects** | | | | | | | | | | | | | | | | | | | | | |
| σ^2^ | 11363.15 | | | 25677.66 | | | 3569.59 | | | 10888.28 | | | 51203.95 | | | 23223.56 | | | 9955.62 | | |
| τ_00_ | 151404.48 _subjCode_ | | | 191840.17 _subjCode_ | | | 5438.67 _subjCode_ | | | 19022.55 _subjCode_ | | | 205765.58 _subjCode_ | | | 58987.17 _subjCode_ | | | 18854.98 _subjCode_ | | |
| ICC | 0.93 | | | 0.88 | | | 0.60 | | | 0.64 | | | 0.80 | | | 0.72 | | | 0.65 | | |
| N | 128 _subjCode_ | | | 128 _subjCode_ | | | 128 _subjCode_ | | | 128 _subjCode_ | | | 128 _subjCode_ | | | 128 _subjCode_ | | | 128 _subjCode_ | | |
| Observations | 253 | | | 252 | | | 250 | | | 254 | | | 253 | | | 253 | | | 254 | | |
| Marginal R^2^ / Conditional R^2^ | 0.137 / 0.940 | | | 0.133 / 0.898 | | | 0.288 / 0.718 | | | 0.180 / 0.702 | | | 0.209 / 0.842 | | | 0.128 / 0.754 | | | 0.194 / 0.721 | | |

**Supplemental Table 5. Group differences in FA.** Statistical results of linear mixed models, showing parameter estimates of FA and standardized beta coefficients as the statistic (also called *t* by lme4). *p*-vals are adjusted for multiple comparisons (Benjamini-Hochberg correction); grey boxes are factors that were marginally significant before correction and non-significant post correction. Green highlights are showing significant factors of group. Model is run as FA*100~hemisphere*group + Age+ Sex + Handedness + (1|subjCode); multiplying FA by 100 allows for more interpretable estimates, otherwise they are all rounded to 0. Statistics are identical with and without multiplier.

|  | **Caudate nucleus FA** | | | **Putamen FA** | | | **Nucleus accumbens FA** | | | **Pallidum FA** | | | **Thalamus FA** | | | **Hippocampus FA** | | | **Amygdala FA** | | |
| --- | --- | --- | --- | --- | --- | --- | --- | --- | --- | --- | --- | --- | --- | --- | --- | --- | --- | --- | --- | --- | --- |
| *Predictors* | *Estimates* | *Statistic* | *p-adj* | *Estimates* | *Statistic* | *p-adj* | *Estimates* | *Statistic* | *p-adj* | *Estimates* | *Statistic* | *p-adj* | *Estimates* | *Statistic* | *p-adj* | *Estimates* | *Statistic* | *p-adj* | *Estimates* | *Statistic* | *p-adj* |
| (Intercept) | 12.83 | 17.44 | **<0.001** | 14.12 | 16.95 | **<0.001** | 16.00 | 13.04 | **<0.001** | 26.05 | 13.15 | **<0.001** | 27.70 | 29.93 | **<0.001** | 13.89 | 10.87 | **<0.001** | 17.56 | 16.08 | **<0.001** |
| hemisphere [Right] | -0.09 | -0.65 | 0.785 | -0.14 | -0.89 | 0.707 | 0.30 | 0.82 | 0.742 | -0.16 | -0.36 | 0.785 | -1.35 | -7.42 | **<0.001** | 0.11 | 0.57 | 0.785 | -0.62 | -2.66 | **0.008** |
| Group [DLD] | -0.59 | -2.51 | **0.047** | 0.11 | 0.41 | 0.785 | -0.15 | -0.35 | 0.785 | -0.27 | -0.41 | 0.785 | -0.88 | -3.02 | **0.013** | 0.14 | 0.37 | 0.785 | -0.18 | -0.50 | 0.621 |
| Age | 0.11 | 1.93 | 0.143 | 0.16 | 2.51 | **0.047** | 0.04 | 0.48 | 0.785 | 0.21 | 1.36 | 0.392 | 0.29 | 4.05 | **<0.001** | 0.21 | 2.09 | 0.113 | 0.10 | 1.15 | 0.249 |
| Sex [Female] | -0.65 | -3.03 | **0.013** | -0.36 | -1.52 | 0.322 | 0.12 | 0.34 | 0.785 | -0.27 | -0.47 | 0.785 | 0.09 | 0.34 | 0.785 | -0.75 | -2.03 | 0.120 | -0.44 | -1.40 | 0.161 |
| Handedness [Left] | -0.31 | -1.06 | 0.569 | 0.23 | 0.71 | 0.785 | 0.39 | 0.80 | 0.742 | -0.58 | -0.76 | 0.756 | 0.78 | 2.16 | 0.100 | 1.23 | 2.45 | 0.051 | 0.25 | 0.59 | 0.557 |
| hemisphere [Right] x Group [DLD] | 0.04 | 0.16 | 0.895 | -0.09 | -0.35 | 0.785 | 0.06 | 0.10 | 0.918 | -0.28 | -0.41 | 0.785 | 0.10 | 0.36 | 0.785 | 0.38 | 1.30 | 0.419 | 0.10 | 0.28 | 0.781 |
| **Random Effects** | | | | | | | | | | | | | | | | | | | | | |
| σ^2^ | 0.77 | | | 0.91 | | | 4.65 | | | 7.12 | | | 1.20 | | | 1.33 | | | 1.96 | | |
| τ_00_ | 0.88 _subjCode_ | | | 1.13 _subjCode_ | | | 1.06 _subjCode_ | | | 5.37 _subjCode_ | | | 1.39 _subjCode_ | | | 3.08 _subjCode_ | | | 1.75 _subjCode_ | | |
| ICC | 0.53 | | | 0.55 | | | 0.19 | | | 0.43 | | | 0.54 | | | 0.70 | | | 0.47 | | |
| N | 127 _subjCode_ | | | 127 _subjCode_ | | | 127 _subjCode_ | | | 127 _subjCode_ | | | 127 _subjCode_ | | | 127 _subjCode_ | | | 127 _subjCode_ | | |
| Observations | 251 | | | 251 | | | 251 | | | 253 | | | 253 | | | 251 | | | 252 | | |
| Marginal R^2^ / Conditional R^2^ | 0.104 / 0.582 | | | 0.060 / 0.581 | | | 0.010 / 0.194 | | | 0.020 / 0.441 | | | 0.267 / 0.660 | | | 0.113 / 0.732 | | | 0.044 / 0.495 | | |

**Supplemental Table 6. Group differences in MD.** Statistical results of linear mixed models, showing parameter estimates of MD and standardized beta coefficients as the statistic (also called *t* by lme4). *p*-vals are adjusted for multiple comparisons (Benjamini-Hochberg correction); grey boxes are factors that were marginally significant before correction and non-significant post correction. ****Model is run as MD*10000~hemisphere*group + Age+ Sex + Handedness + (1|subjCode); multiplying MD by 10000 allows for more interpretable estimates, otherwise they are all rounded to 0. Statistics are identical with and without multiplier

|  | **Caudate nucleus MD** | | | **Putamen MD** | | | **Nucleus accumbens MD** | | | **Pallidum MD** | | | **Thalamus MD** | | | **Hippocampus MD** | | | **Amygdala MD** | | |
| --- | --- | --- | --- | --- | --- | --- | --- | --- | --- | --- | --- | --- | --- | --- | --- | --- | --- | --- | --- | --- | --- |
| *Predictors* | *Estimates* | *Statistic* | *p* | *Estimates* | *Statistic* | *p* | *Estimates* | *Statistic* | *p* | *Estimates* | *Statistic* | *p* | *Estimates* | *Statistic* | *p* | *Estimates* | *Statistic* | *p* | *Estimates* | *Statistic* | *p* |
| (Intercept) | 0.08 | 27.80 | **<0.001** | 0.08 | 108.42 | **<0.001** | 0.08 | 51.29 | **<0.001** | 0.08 | 62.25 | **<0.001** | 0.08 | 69.34 | **<0.001** | 0.09 | 26.83 | **<0.001** | 0.09 | 48.58 | **<0.001** |
| hemisphere [Right] | -0.00 | -1.95 | 0.146 | 0.00 | 6.30 | **<0.001** | 0.00 | 5.33 | **<0.001** | 0.00 | 0.66 | 0.713 | 0.00 | 3.31 | **0.005** | -0.00 | -1.22 | 0.448 | -0.00 | -2.43 | 0.057 |
| Group [DLD] | -0.00 | -0.32 | 0.839 | -0.00 | -0.54 | 0.759 | 0.00 | 0.21 | 0.869 | -0.00 | -0.59 | 0.735 | 0.00 | 0.50 | 0.774 | 0.00 | 0.83 | 0.641 | 0.00 | 0.14 | 0.911 |
| Age | -0.00 | -1.11 | 0.485 | -0.00 | -8.19 | **<0.001** | -0.00 | -0.78 | 0.665 | -0.00 | -5.50 | **<0.001** | -0.00 | -2.17 | 0.106 | 0.00 | 1.73 | 0.211 | -0.00 | -1.06 | 0.512 |
| Sex [Female] | -0.00 | -1.59 | 0.262 | -0.00 | -3.92 | **0.001** | -0.00 | -0.37 | 0.839 | -0.00 | -1.82 | 0.182 | -0.00 | -0.62 | 0.730 | 0.00 | 0.27 | 0.854 | -0.00 | -1.58 | 0.262 |
| Handedness [Left] | 0.00 | 1.19 | 0.448 | -0.00 | -1.00 | 0.536 | -0.00 | -0.34 | 0.839 | 0.00 | 0.42 | 0.831 | 0.00 | 0.71 | 0.713 | 0.00 | 1.98 | 0.146 | 0.00 | 1.38 | 0.363 |
| hemisphere [Right] x Group [DLD] | 0.00 | 0.67 | 0.713 | -0.00 | -0.07 | 0.948 | -0.00 | -0.98 | 0.536 | -0.00 | -0.34 | 0.839 | 0.00 | 1.19 | 0.448 | 0.00 | 1.97 | 0.146 | 0.00 | 0.23 | 0.869 |
| **Random Effects** | | | | | | | | | | | | | | | | | | | | | |
| σ^2^ | 0.00 | | | 0.00 | | | 0.00 | | | 0.00 | | | 0.00 | | | 0.00 | | | 0.00 | | |
| τ_00_ | 0.00 _subjCode_ | | | 0.00 _subjCode_ | | | 0.00 _subjCode_ | | | 0.00 _subjCode_ | | | 0.00 _subjCode_ | | | 0.00 _subjCode_ | | | 0.00 _subjCode_ | | |
| ICC | 0.22 | | | 0.68 | | | 0.38 | | | 0.62 | | | 0.44 | | | 0.57 | | | 0.26 | | |
| N | 127 _subjCode_ | | | 127 _subjCode_ | | | 127 _subjCode_ | | | 127 _subjCode_ | | | 127 _subjCode_ | | | 127 _subjCode_ | | | 127 _subjCode_ | | |
| Observations | 250 | | | 256 | | | 251 | | | 253 | | | 251 | | | 248 | | | 251 | | |
| Marginal R^2^ / Conditional R^2^ | 0.043 / 0.253 | | | 0.387 / 0.805 | | | 0.091 / 0.434 | | | 0.181 / 0.687 | | | 0.105 / 0.496 | | | 0.062 / 0.596 | | | 0.058 / 0.303 | | |

**Supplemental Table 7. Group differences in f_iso_.** Statistical results of linear mixed models evaluating group differences of NODDI parameter – f_iso_. Table shows parameter estimates and standardized beta coefficients as the statistic (also called t by lme4). Green highlights are showing significant factors of group. ****Model is run as fiso*100~hemisphere*group + Age+ Sex + Handedness + (1|subjCode); multiplying 100 allows for more interpretable estimates, otherwise they are all rounded to 0. Statistics are identical with and without multiplier. These are uncorrected for multiple comparisons.

|  | **Caudate nucleus fiso** | | | **Putamen fiso** | | | **Nucleus accumbens fiso** | | | **Pallidum fiso** | | | **Thalamus fiso** | | | **Hippocampus fiso** | | | **Amygdala fiso** | | |
| --- | --- | --- | --- | --- | --- | --- | --- | --- | --- | --- | --- | --- | --- | --- | --- | --- | --- | --- | --- | --- | --- |
| *Predictors* | *Estimates* | *Statistic* | *p* | *Estimates* | *Statistic* | *p* | *Estimates* | *Statistic* | *p* | *Estimates* | *Statistic* | *p* | *Estimates* | *Statistic* | *p* | *Estimates* | *Statistic* | *p* | *Estimates* | *Statistic* | *p* |
| (Intercept) | 5.67 | 2.05 | **0.041** | 1.40 | 7.84 | **<0.001** | 2.27 | 3.10 | **0.002** | 3.61 | 5.36 | **<0.001** | 3.39 | 3.16 | **0.002** | 7.34 | 4.20 | **<0.001** | 3.74 | 3.27 | **0.001** |
| hemisphere [Right] | -2.85 | -4.17 | **<0.001** | 0.04 | 1.68 | 0.094 | 0.18 | 1.08 | 0.283 | 0.28 | 2.49 | **0.013** | -0.06 | -0.41 | 0.686 | 0.31 | 0.90 | 0.368 | 0.00 | 0.01 | 0.989 |
| Group [DLD] | 1.32 | 1.42 | 0.157 | 0.03 | 0.47 | 0.642 | 0.39 | 1.60 | 0.112 | -0.32 | -1.55 | 0.123 | 0.66 | 2.07 | **0.040** | 1.34 | 2.45 | **0.015** | 0.32 | 0.86 | 0.388 |
| Age | 0.28 | 1.30 | 0.194 | 0.04 | 2.77 | **0.006** | 0.04 | 0.73 | 0.466 | 0.23 | 4.38 | **<0.001** | 0.28 | 3.45 | **0.001** | 0.42 | 3.15 | **0.002** | 0.18 | 2.07 | **0.040** |
| Sex [Female] | 0.24 | 0.30 | 0.766 | -0.12 | -2.29 | **0.023** | -0.00 | -0.00 | 0.999 | -0.45 | -2.31 | **0.022** | -0.06 | -0.19 | 0.851 | -0.06 | -0.12 | 0.908 | -0.19 | -0.58 | 0.564 |
| Handedness [Left] | 1.42 | 1.31 | 0.193 | 0.08 | 1.13 | 0.261 | 0.52 | 1.84 | 0.068 | -0.65 | -2.48 | **0.014** | 0.56 | 1.31 | 0.190 | 0.71 | 1.05 | 0.296 | 0.29 | 0.64 | 0.525 |
| hemisphere [Right] x Group [DLD] | 0.18 | 0.17 | 0.862 | -0.00 | -0.02 | 0.988 | 0.02 | 0.10 | 0.923 | -0.15 | -0.86 | 0.390 | 0.17 | 0.69 | 0.491 | -0.26 | -0.49 | 0.627 | 0.19 | 0.53 | 0.598 |
| **Random Effects** | | | | | | | | | | | | | | | | | | | | | |
| σ^2^ | 16.66 | | | 0.03 | | | 1.00 | | | 0.48 | | | 0.94 | | | 4.28 | | | 1.92 | | |
| τ_00_ | 9.21 _subjCode_ | | | 0.06 _subjCode_ | | | 0.73 _subjCode_ | | | 0.82 _subjCode_ | | | 2.10 _subjCode_ | | | 4.80 _subjCode_ | | | 2.05 _subjCode_ | | |
| ICC | 0.36 | | | 0.71 | | | 0.42 | | | 0.63 | | | 0.69 | | | 0.53 | | | 0.52 | | |
| N | 126 _subjCode_ | | | 126 _subjCode_ | | | 126 _subjCode_ | | | 126 _subjCode_ | | | 126 _subjCode_ | | | 126 _subjCode_ | | | 126 _subjCode_ | | |
| Observations | 248 | | | 254 | | | 248 | | | 255 | | | 251 | | | 250 | | | 252 | | |
| Marginal R^2^ / Conditional R^2^ | 0.098 / 0.419 | | | 0.099 / 0.737 | | | 0.047 / 0.451 | | | 0.185 / 0.699 | | | 0.112 / 0.726 | | | 0.091 / 0.571 | | | 0.040 / 0.536 | | |

**Supplemental Table 8. Group differences in f_intra._** Statistical results of linear mixed models evaluating group differences of NODDI parameter – f_intra_. Table shows parameter estimates and standardized beta coefficients as the statistic (also called t by lme4). Green highlights are showing significant factors of group. ****Model is run as fintra*100~hemisphere*group + Age+ Sex + Handedness + (1|subjCode); multiplying by 100 allows for more interpretable estimates, otherwise they are all rounded to 0. Statistics are identical with and without multiplier. These are uncorrected for multiple comparisons.

|  | **Caudate nucleus fintra** | | | **Putamen fintra** | | | **Nucleus accumbens fintra** | | | **Pallidum fintra** | | | **Thalamus fintra** | | | **Hippocampus fintra** | | | **Amygdala fintra** | | |
| --- | --- | --- | --- | --- | --- | --- | --- | --- | --- | --- | --- | --- | --- | --- | --- | --- | --- | --- | --- | --- | --- |
| *Predictors* | *Estimates* | *Statistic* | *p* | *Estimates* | *Statistic* | *p* | *Estimates* | *Statistic* | *p* | *Estimates* | *Statistic* | *p* | *Estimates* | *Statistic* | *p* | *Estimates* | *Statistic* | *p* | *Estimates* | *Statistic* | *p* |
| (Intercept) | 43.18 | 38.11 | **<0.001** | 43.46 | 63.28 | **<0.001** | 39.70 | 44.69 | **<0.001** | 45.56 | 27.49 | **<0.001** | 45.26 | 44.48 | **<0.001** | 36.44 | 38.91 | **<0.001** | 38.91 | 41.13 | **<0.001** |
| hemisphere [Right] | -1.49 | -7.49 | **<0.001** | -0.67 | -7.62 | **<0.001** | -1.36 | -7.59 | **<0.001** | 0.15 | 0.69 | 0.490 | -1.22 | -7.63 | **<0.001** | -0.22 | -1.59 | 0.113 | -0.19 | -1.10 | 0.274 |
| Group [DLD] | 0.54 | 1.52 | 0.129 | 0.07 | 0.36 | 0.722 | 0.07 | 0.24 | 0.812 | -0.13 | -0.26 | 0.793 | -0.06 | -0.20 | 0.839 | 0.33 | 1.18 | 0.240 | 0.02 | 0.06 | 0.950 |
| Age | 0.30 | 3.40 | **0.001** | 0.47 | 8.87 | **<0.001** | 0.10 | 1.46 | 0.147 | 0.94 | 7.40 | **<0.001** | 0.47 | 6.04 | **<0.001** | 0.23 | 3.22 | **0.001** | 0.20 | 2.71 | **0.007** |
| Sex [Female] | 0.54 | 1.66 | 0.098 | 0.48 | 2.39 | **0.017** | -0.22 | -0.86 | 0.390 | 0.15 | 0.32 | 0.749 | 0.07 | 0.25 | 0.802 | -0.19 | -0.71 | 0.481 | 0.14 | 0.51 | 0.612 |
| Handedness [Left] | 0.50 | 1.12 | 0.265 | 0.41 | 1.54 | 0.125 | 0.31 | 0.90 | 0.370 | -1.03 | -1.59 | 0.113 | 0.52 | 1.32 | 0.189 | 0.50 | 1.38 | 0.170 | -0.07 | -0.20 | 0.841 |
| hemisphere [Right] x Group [DLD] | -0.29 | -0.93 | 0.354 | 0.05 | 0.40 | 0.686 | 0.03 | 0.12 | 0.907 | -0.03 | -0.10 | 0.920 | 0.24 | 0.95 | 0.342 | -0.13 | -0.61 | 0.544 | 0.02 | 0.07 | 0.945 |
| **Random Effects** | | | | | | | | | | | | | | | | | | | | | |
| σ^2^ | 1.44 | | | 0.28 | | | 1.17 | | | 1.64 | | | 0.95 | | | 0.70 | | | 1.03 | | |
| τ_00_ | 2.27 _subjCode_ | | | 0.97 _subjCode_ | | | 1.25 _subjCode_ | | | 5.61 _subjCode_ | | | 1.95 _subjCode_ | | | 1.71 _subjCode_ | | | 1.58 _subjCode_ | | |
| ICC | 0.61 | | | 0.78 | | | 0.52 | | | 0.77 | | | 0.67 | | | 0.71 | | | 0.60 | | |
| N | 128 _subjCode_ | | | 128 _subjCode_ | | | 128 _subjCode_ | | | 128 _subjCode_ | | | 128 _subjCode_ | | | 128 _subjCode_ | | | 128 _subjCode_ | | |
| Observations | 250 | | | 255 | | | 252 | | | 253 | | | 254 | | | 255 | | | 252 | | |
| Marginal R^2^ / Conditional R^2^ | 0.216 / 0.695 | | | 0.404 / 0.867 | | | 0.176 / 0.603 | | | 0.292 / 0.840 | | | 0.262 / 0.758 | | | 0.092 / 0.736 | | | 0.050 / 0.624 | | |

**Supplemental Table 9. Group differences in OD.** Statistical results of linear mixed models evaluating group differences of NODDI parameter – OD. Table shows parameter estimates and standardized beta coefficients as the statistic (also called t by lme4). Green highlights are showing significant factors of group. ****Model is run as OD*100~hemisphere*group + Age+ Sex + Handedness + (1|subjCode); multiplying by 100 allows for more interpretable estimates, otherwise they are all rounded to 0. Statistics are identical with and without multiplier. These are uncorrected for multiple comparisons.

|  | **Caudate nucleus OD** | | | **Putamen OD** | | | **Nucleus accumbens OD** | | | **Pallidum OD** | | | **Thalamus OD** | | | **Hippocampus OD** | | | **Amygdala OD** | | |
| --- | --- | --- | --- | --- | --- | --- | --- | --- | --- | --- | --- | --- | --- | --- | --- | --- | --- | --- | --- | --- | --- |
| *Predictors* | *Estimates* | *Statistic* | *p* | *Estimates* | *Statistic* | *p* | *Estimates* | *Statistic* | *p* | *Estimates* | *Statistic* | *p* | *Estimates* | *Statistic* | *p* | *Estimates* | *Statistic* | *p* | *Estimates* | *Statistic* | *p* |
| (Intercept) | 51.51 | 28.12 | **<0.001** | 49.31 | 33.65 | **<0.001** | 44.39 | 23.99 | **<0.001** | 34.93 | 17.99 | **<0.001** | 31.87 | 44.44 | **<0.001** | 43.47 | 23.51 | **<0.001** | 41.90 | 27.28 | **<0.001** |
| hemisphere [Right] | -0.52 | -2.07 | **0.039** | -0.18 | -0.86 | 0.392 | -1.24 | -2.51 | **0.013** | -1.05 | -2.29 | **0.023** | 0.38 | 4.62 | **<0.001** | -0.20 | -0.73 | 0.466 | 1.14 | 3.32 | **0.001** |
| Group [DLD] | 0.66 | 1.20 | 0.232 | 0.17 | 0.39 | 0.696 | 0.69 | 1.07 | 0.284 | 0.35 | 0.55 | 0.583 | 0.69 | 3.25 | **0.001** | -0.04 | -0.08 | 0.938 | -0.23 | -0.46 | 0.647 |
| Age | -0.13 | -0.90 | 0.367 | 0.01 | 0.12 | 0.903 | 0.12 | 0.82 | 0.411 | 0.31 | 2.08 | **0.038** | -0.14 | -2.46 | **0.015** | -0.29 | -2.04 | **0.042** | -0.08 | -0.66 | 0.507 |
| Sex [Female] | 0.72 | 1.36 | 0.176 | 0.83 | 1.98 | **0.049** | -0.34 | -0.65 | 0.518 | 0.69 | 1.24 | 0.215 | 0.12 | 0.56 | 0.575 | 0.91 | 1.72 | 0.087 | 0.74 | 1.69 | 0.092 |
| Handedness [Left] | -1.20 | -1.65 | 0.100 | -0.05 | -0.09 | 0.927 | -0.74 | -1.04 | 0.299 | 0.80 | 1.08 | 0.283 | -1.04 | -3.70 | **<0.001** | -1.52 | -2.09 | **0.037** | -0.53 | -0.89 | 0.377 |
| hemisphere [Right] x  Group [DLD] | 0.23 | 0.59 | 0.556 | 0.07 | 0.22 | 0.827 | -0.09 | -0.12 | 0.901 | 0.68 | 0.95 | 0.342 | -0.21 | -1.63 | 0.104 | -0.74 | -1.73 | 0.085 | 0.58 | 1.10 | 0.274 |
| **Random Effects** | | | | | | | | | | | | | | | | | | | | | |
| σ^2^ | 2.30 | | | 1.57 | | | 8.95 | | | 7.62 | | | 0.25 | | | 2.81 | | | 4.16 | | |
| τ_00_ | 6.75 _subjCode_ | | | 4.05 _subjCode_ | | | 3.38 _subjCode_ | | | 4.68 _subjCode_ | | | 1.08 _subjCode_ | | | 6.50 _subjCode_ | | | 3.29 _subjCode_ | | |
| ICC | 0.75 | | | 0.72 | | | 0.27 | | | 0.38 | | | 0.81 | | | 0.70 | | | 0.44 | | |
| N | 127 _subjCode_ | | | 127 _subjCode_ | | | 127 _subjCode_ | | | 127 _subjCode_ | | | 127 _subjCode_ | | | 127 _subjCode_ | | | 127 _subjCode_ | | |
| Observations | 253 | | | 251 | | | 253 | | | 251 | | | 251 | | | 251 | | | 249 | | |
| Marginal R^2^ / Conditional R^2^ | 0.058 / 0.761 | | | 0.029 / 0.728 | | | 0.051 / 0.311 | | | 0.051 / 0.412 | | | 0.186 / 0.849 | | | 0.095 / 0.727 | | | 0.087 / 0.490 | | |


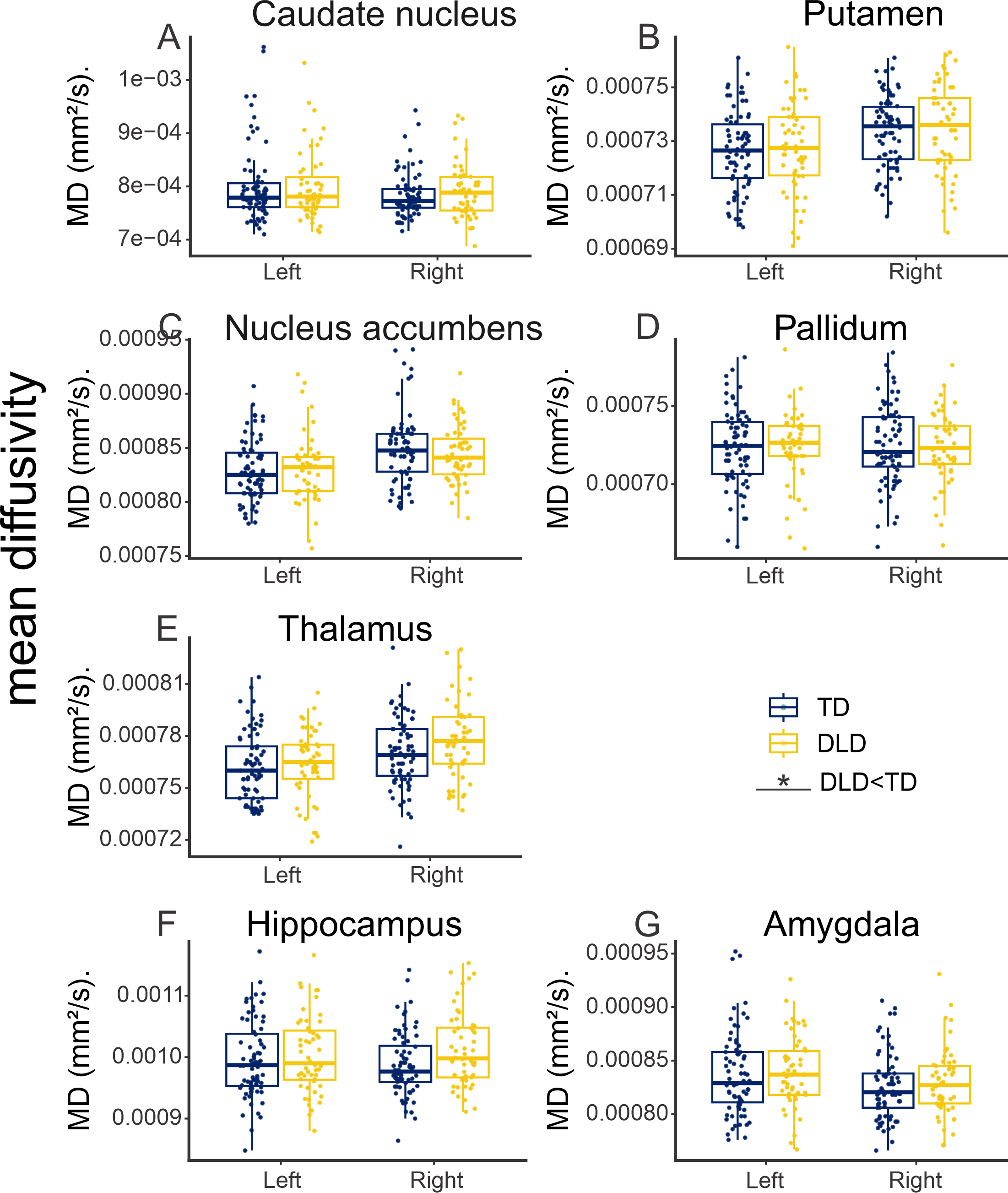


Supplemental Figure 1. Mean diffusivity (MD) by region, group, and hemisphere. No significant group differences based on linear mixed models (full statistical tables are in Supplemental Table 6). Boxplot shows first quartile, median, and third quartile; whiskers show first and third quartile ±1.5*interquartile range.. Each individual data point is mean diffusivity of one participant’s subcortical structure. For all plots, N=126-128 participants (outliers removed as discussed in Methods). Abbrev: DLD=developmental language disorder; TD = typically developing; MD = mean diffusivity


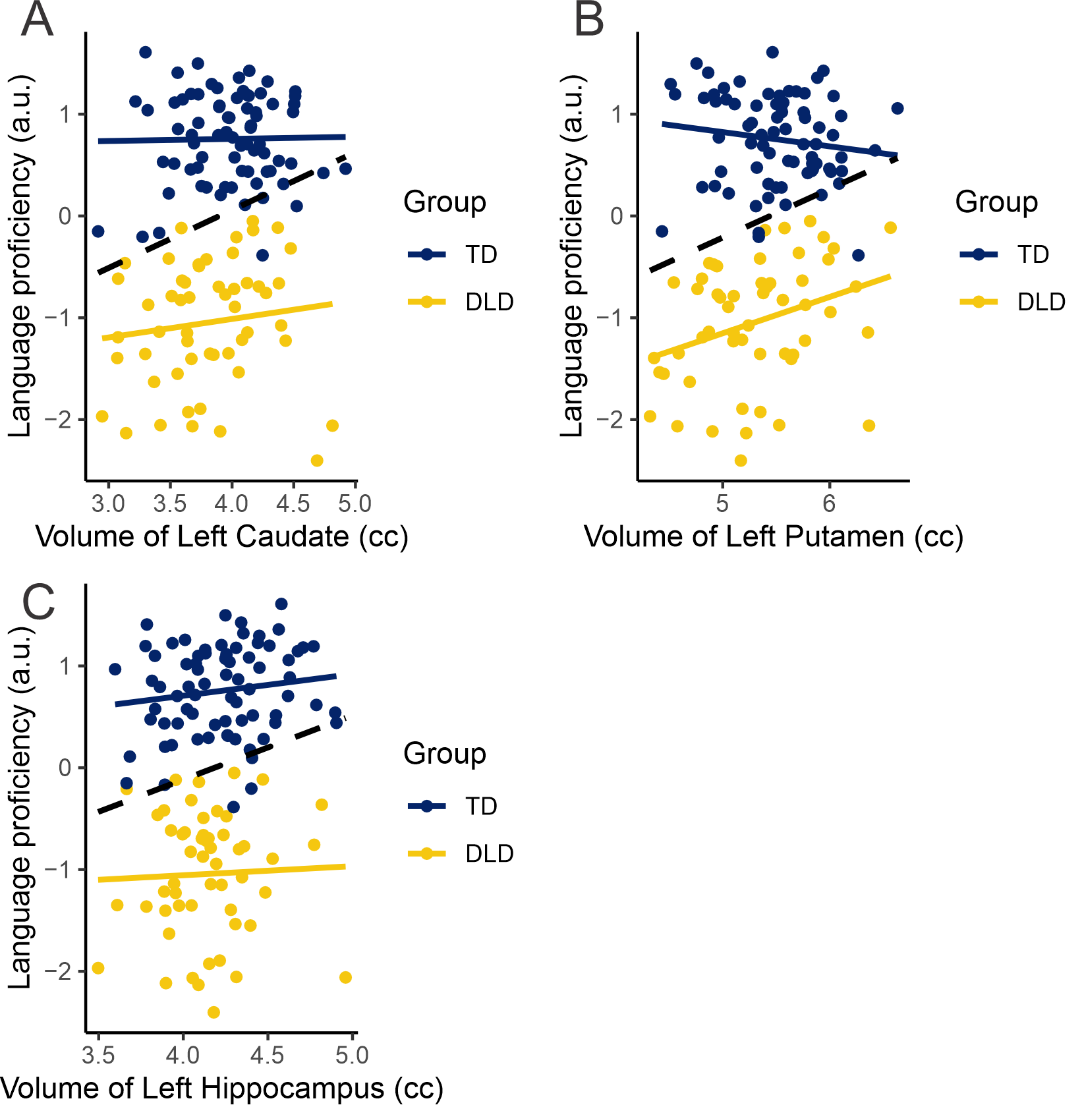


Supplemental Figure 2. Selected subcortical volumes versus language proficiency (summary measure derived from language tasks, arbitrary units). Each individual data marker is the volume of one structure from one participant’s subcortical structure. Lines are best fit within group (yellow, blue) and between groups (dashed black). For all plots, N=126-128 participants (outliers removed as discussed in Methods). Lines are best fit; no statistical tests applied. Abbrev: DLD=developmental language disorder; TD = typically developing.


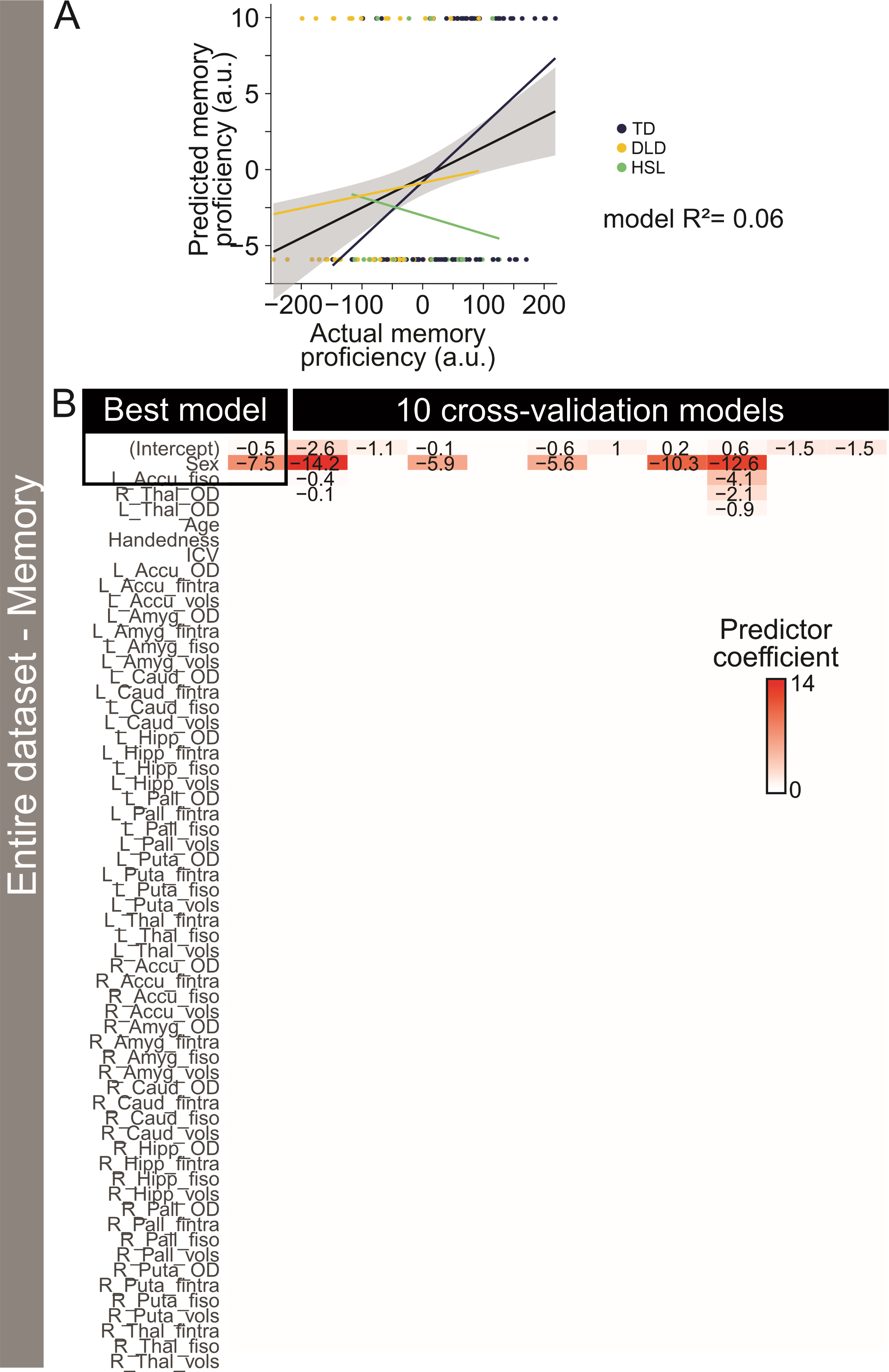


Supplemental Figure 3. Results of multivariate analyses predicting **memory** factor across entire cohort. The model accounted for R^2^=0.06. (A) shows actual language proficiency on x axis and predicted language proficiency on y axis (both arbitrary units), where each dot is one participant (N=156). The model fit is shown within (blue, yellow, green) and across (black) groups. B shows the predictors selected by machine learning algorithm across all participants in the model (highlighted in black box) and then each of 10 cross-validation runs with 90% of the data at a time, in order to show the stability of the predictors. Abbrev: DLD = developmental language disorder; TD = typically developing; HSL = history of speech and language concerns

**References:**

Badcock, N. A., Bishop, D. V. M., Hardiman, M. J., Barry, J. G., & Watkins, K. E. (2012). Co-localisation of abnormal brain structure and function in specific language impairment. *Brain and Language*, *120*(3), 310–320. https://doi.org/10.1016/j.bandl.2011.10.006

Girbau-Massana, D., Garcia-Marti, G., Marti-Bonmati, L., & Schwartz, R. G. (2014). Gray-white matter and cerebrospinal fluid volume differences in children with Specific Language Impairment and/or Reading Disability. *Neuropsychologia*, *56*(1), 90–100. https://doi.org/10.1016/j.neuropsychologia.2014.01.004

Herbert, M. R., Ziegler, D. A., Makris, N., Bakardjiev, A., Hodgson, J., Adrien, K. T., Kennedy, D. N., Filipek, P. A., & Caviness, V. S. (2003). Larger brain and white matter volumes in children with developmental language disorder. *Developmental Science*, *6*(4), F11–F22. https://doi.org/10.1111/1467-7687.00291

Jernigan, T. L., Hesselink, J. R., Sowell, E., & Tallal, P. A. (1991). Cerebral Structure on Magnetic Resonance Imaging in Language- and Learning-Impaired Children. *Archives of Neurology*, *48*(5), 539–545. https://doi.org/10.1001/ARCHNEUR.1991.00530170103028

Lee, J. C., Nopoulos, P. C., & Bruce Tomblin, J. (2013). Abnormal subcortical components of the corticostriatal system in young adults with DLI: a combined structural MRI and DTI study. *Neuropsychologia*, *51*(11), 2154–2161. https://doi.org/10.1016/j.neuropsychologia.2013.07.011

Pigdon, L., Willmott, C., Reilly, S., Conti-Ramsden, G., Gaser, C., Connelly, A., & Morgan, A. T. (2019). Grey matter volume in developmental speech and language disorder. *Brain Structure and Function*, *224*(9), 3387–3398. https://doi.org/10.1007/s00429-019-01978-7

Soriano-Mas, C., Pujol, J., Ortiz, H., Deus, J., López-Sala, A., & Sans, A. (2009). Age-related brain structural alterations in children with specific language impairment. *Human Brain Mapping*, *30*(5), 1626–1636. https://doi.org/10.1002/hbm.20620
